# Prodrugs of NSAIDs: A Review

**DOI:** 10.2174/1874104501711010146

**Published:** 2017-11-30

**Authors:** Kamal Shah, Jeetendra K. Gupta, Nagendra S. Chauhan, Neeraj Upmanyu, Sushant K. Shrivastava, Pradeep Mishra

**Affiliations:** 1 Institute of Pharmaceutical Research, GLA University, Mathura, U.P.- 281406, India; 2 School of Pharmacy & Research, Peoples University, Bhopal, M.P.- 462037, India; 3 Department of Pharmaceutics, Institute of Technology, Banaras Hindu University, Varanasi U.P.- 221005, India; 4 Drugs Testing Laboratory Avam Anusandhan Kendra,Raipur (CG),India

**Keywords:** Prodrug, Synergistic, NSAIDs, Ulcergenocity, Gastrointestinal toxicity, Enzymatic attack

## Abstract

**Intoroduction:**

Prodrug approach deals with chemical biotransformation or enzymatic conversion or involves inactive or less active bio-reversible derivatives of active drug molecules. They have to pass through enzymatic or chemical biotransformation before eliciting their pharmacological action.

**Methods & Materials:**

The two different pharmacophores combine to give synergistic activity or may help in targeting the active drug to its target. Prodrug super seeds the problems of prodrug designing, for example solubility enhancement, bioavailability enhancement, chemical stability improvement, presystemic metabolism, site specific delivery, toxicity masking, improving patient acceptance, or eradicating undesirable adverse effects.

**Results:**

As an outcome the search for a prodrug or mutual prodrug with reduced toxicity has continued during recent years. This present review emphasizes the common help to revamp physiochemical, pharmaceutical and therapeutic effectiveness of drugs.

**Conclusion:**

This gives the researcher a common platform where they can find prodrugs of commonly used NSAIDs to overcome the gastrointestinal toxicity (irritation, ulcergenocity and bleeding).

## 1 INTRODUCTION

The term prodrug was first coined by Albert [1]. According to him, a pharmacologically slothful compound that is modified by the mammalian system into an active substance by either chemical or metabolic means. This action is known as drug latentiation, which is chemical moderation of a biologically active compound to form a parent compound that undergoes *in vivo* enzymatic attack [2]. Prodrugs are said to be bioreversible derivatives which on biotransformation change into active pharmacophore.

Prodrug approach has a wide range of applications. It has significant role in drug designing. A lot of scientists designed various prodrugs with different objectivity like correction of pharmacokinetics parameters, improvement of organoleptic properties or chemical properties. Following are some examples taken from Notari [3] (Table **1**).

## 2 CLASSIFICATION OF PRODRUG

**Fig. (1):**
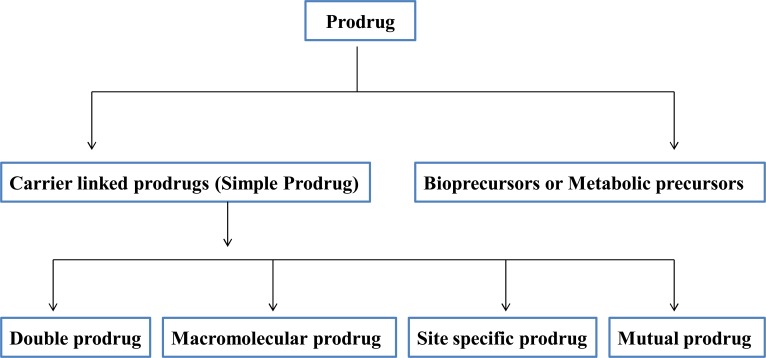
Classification of prodrug.

Broadly prodrugs can be classified in two types:

1. Carrier linked prodrugs
2. Bioprecursors (Fig. **1**)

### 2.1 Carrier Linked Prodrugs (Simple Prodrug)

**Fig. (2):**
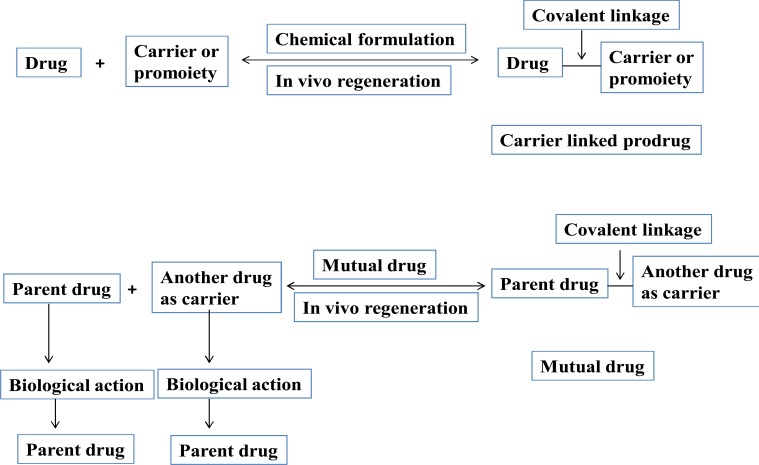
Schematic representation of carrier-linked prodrug and mutual prodrug.

Carrier linked prodrug has an inert carrier or transport which is coupled covalently with active drug. They have ester or amide linkage. They got biotransformed chemically or enzymatically and release the active drug. The carrier-linked prodrugs should be non-toxic. They should mask the unwanted side effects. They alter the physiochemical properties of active drug (Fig. **2**). On the basis of carrier used, further it can be classified as follows:

**a) Pro-prodrug**

**Fig. (3):**
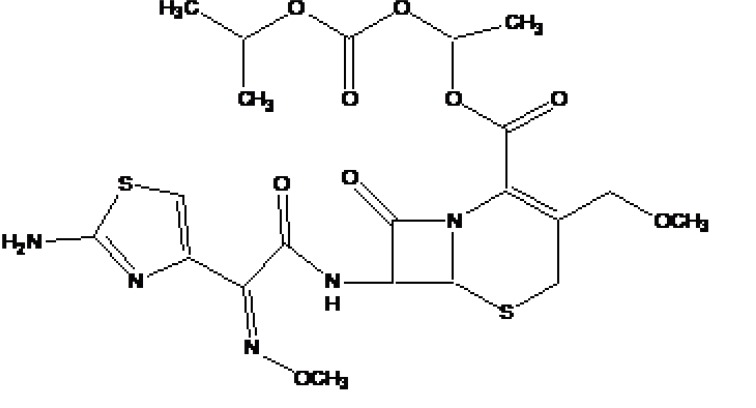
Cefpodoxime proxetil.

It is a prodrug, where drug is derivatized in such a way that conversion through enzymes is possible before the latter can break to release the active drug for example Cefpodoxime proxetil (Fig. **3**).

**b) Macromolecular prodrug**

**Fig. (4):**
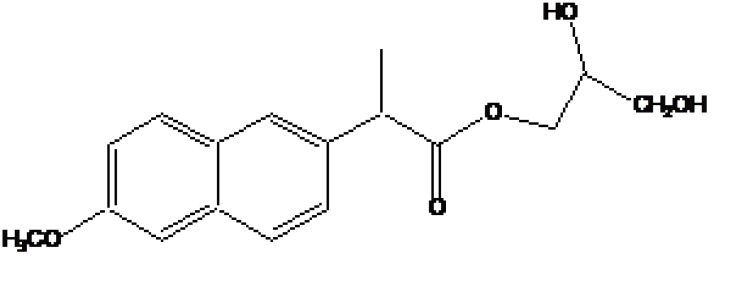
Naproxen-2-glyceride.

The carrier used here are large molecular weight compounds for example: polysaccharides, cyclodextrins, polymers and proteins, *e.g*. Naproxen-2-glyceride (Fig. **4**).

**c) Site specific prodrug**

**Fig. (5):**
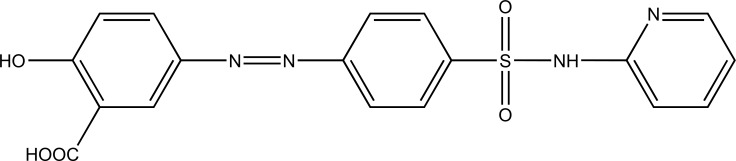
Sulfasalazine.

Such prodrug is used for targeting the active drug at specific site, *e.g*. sulfasalazine (Fig. **5**) which consists of 5-aminosalicylic acid and sulfapyridine. Both are pharmacologically active agents, linked by azo linkage. The 5-aminosalicylic acid is released in the colon. The advantage of this approach is it to release the drug in required concentration at the active site.

**d) Mutual prodrug**

**Fig. (6):**
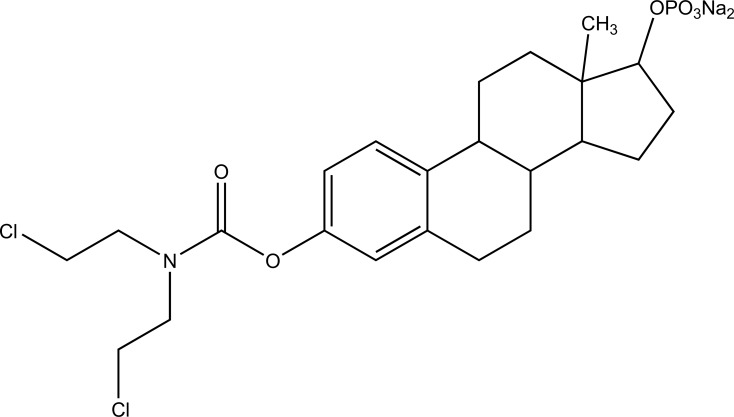
Estramustine.

It consists of two pharmacologically active drugs joined with each other. They are taken together with the aim to mask the side effects of active drug and give the synergistic action. For example Estramustine (Fig. **6**) has a phosphorylated steroid (17- α- estradiol) coupled to Nor-mustard which has carbamate linkage (Fig. **2**).

### 2.2 Bioprecursors

**Fig. (7):**
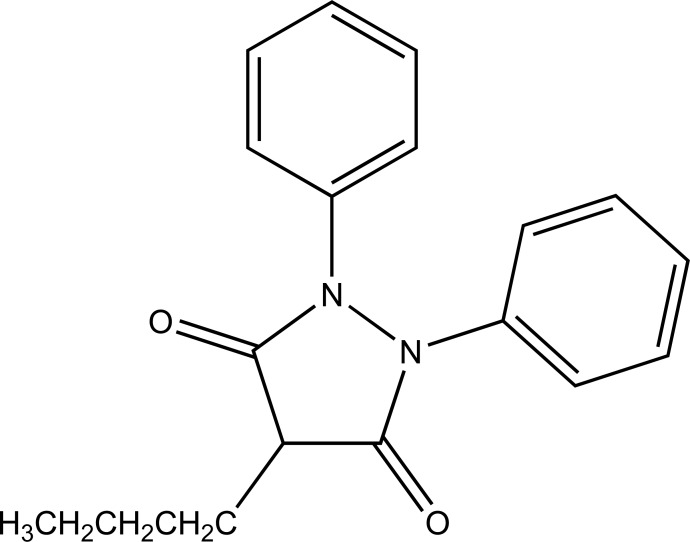
Phenylbutazone.

Here parent drug is obtained by redox transformation through enzymes. Her prodrug result by chemical modification of parent drug. The lipophilicity does not alter generally. For example phenylbutazone (Fig. **7**) which is a metabolic precursor prodrug of oxyphenbutazone.

## 3 PREREQUISITES OF IDEAL PRODRUG

The prodrugs should have following features:

1. It should be pharmacological inert.
2. It should have fast transformation, by chemicals or enzymes.
3. It should be non-toxic metabolic component.

The main objectives of a prodrug designing are, to have active drugs to their active sites, to show the required pharmacological effects while minimizing adverse effects, to get the desired clinical and therapeutic activity of those drugs which have some undesirable properties, and to prevent the co-administration of two drugs so that the same pharmacological activity obtained with minimum side effects. Co administration does not always confirm equivalent absorption or transportation to desired site. So, mutual prodrug concept is fruitful when two pharmacologically active drugs are administered at similar time. Mutual prodrugs were prepared by keeping the objectivity of improving drug's efficacy, optimizing delivery and lowering toxicities.

## 4 GENERAL APPLICATIONS OF PRODRUG APPROACHES

Prodrugs are designed to eradicate the problems faced by parent drug. They are made to alter pharmacokinetics parameters or to alter the physicochemical properties. The purpose of prodrug design may vary [4-6]. A real prodrug has chemical stability when formulated in suitable dosage form, should release the drug at targeted site, the promoiety should be non toxic [7].

Drug design scientist widely uses prodrug approach for developing new molecule. Few examples are reported as illustrated in Table **1**.

## 5 PHARMACEUTICAL APPLICATION

The prodrug approach is used to resolve the undesirable properties and physicochemical problems accompanied by active drug [8].

**Fig. (8):**
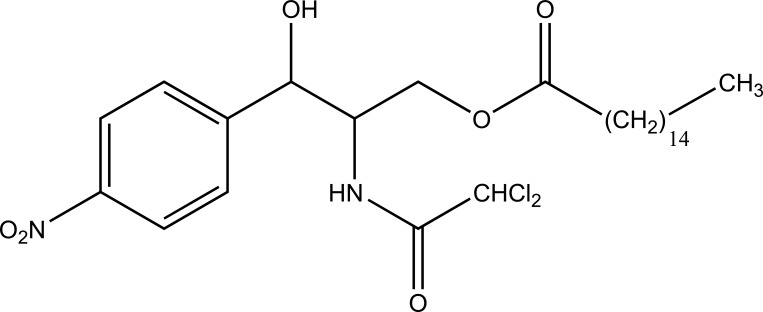
Chloromphenicol Palmitate.

**Fig. (9):**
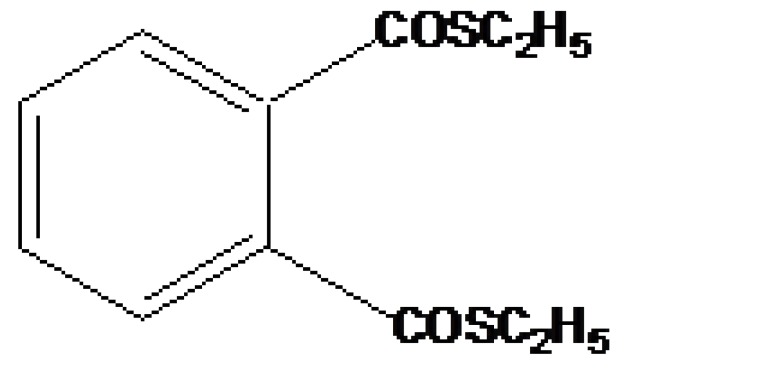
Ethylmercaptan.

**Fig. (10):**
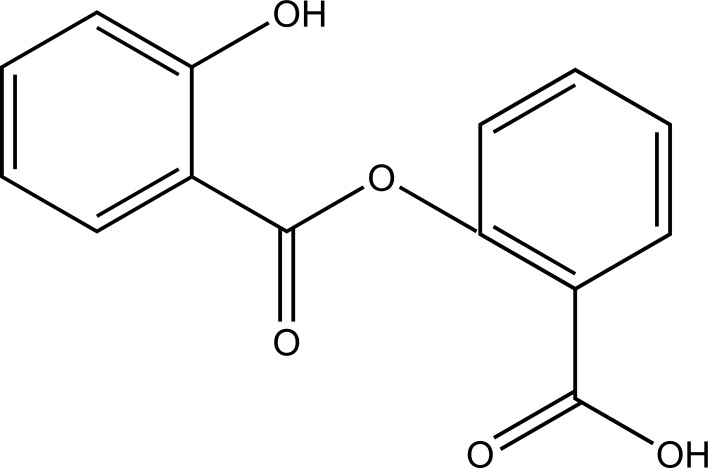
Salsalate.

**Fig. (11):**
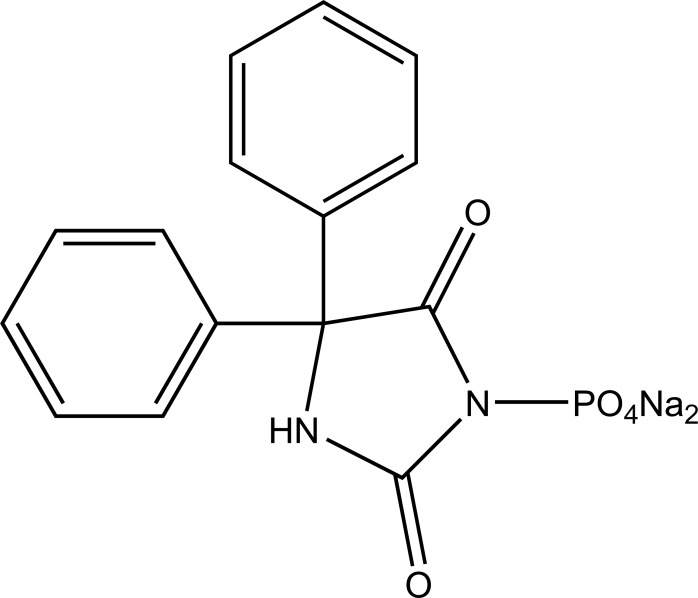
Prodrug of Phenytoin.

**Fig. (12):**
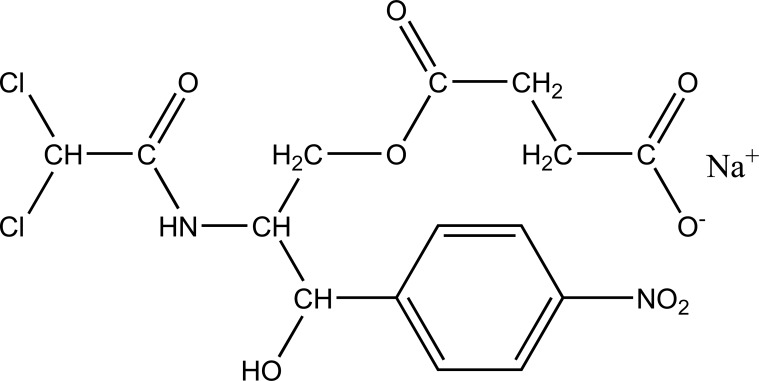
Chloromphenicaol Sodium Succinate.

**Fig. (13):**
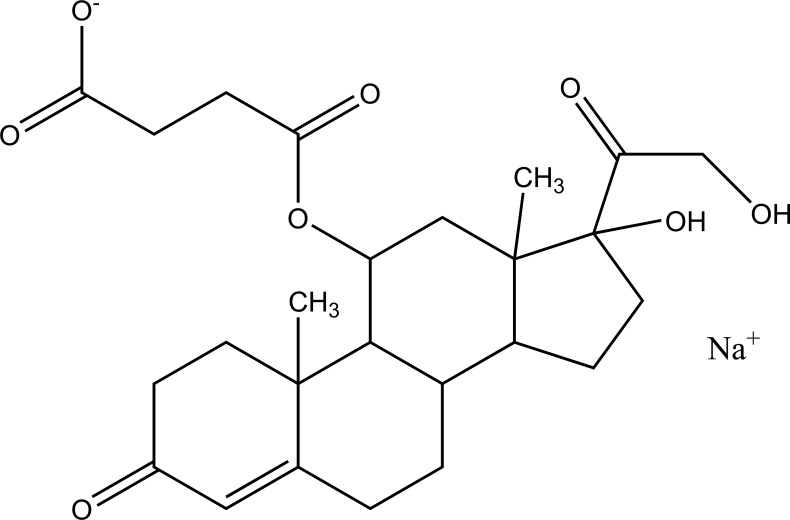
Cortisol.

**Fig. (14):**
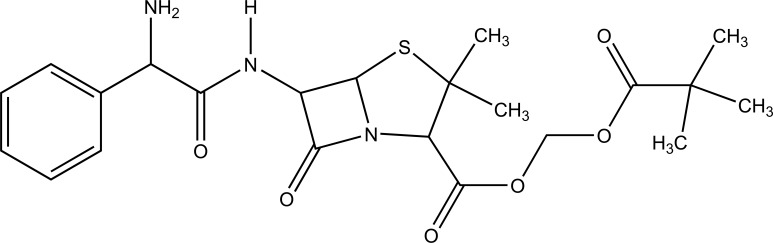
Pivampicillin.

**Fig. (15):**
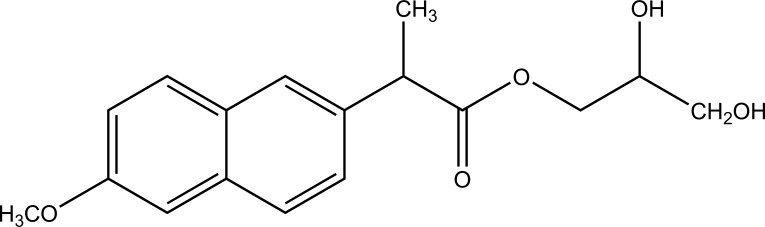
Glycerol Ester of Naproxen.

**Fig. (16):**
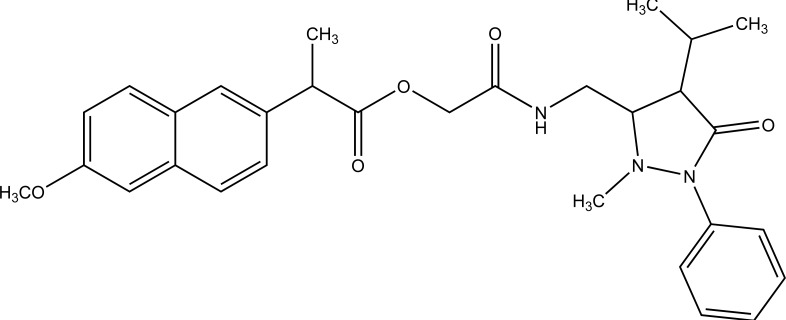
Naproxen-propyphenazone mutual prodrugs.

**Fig. (17):**
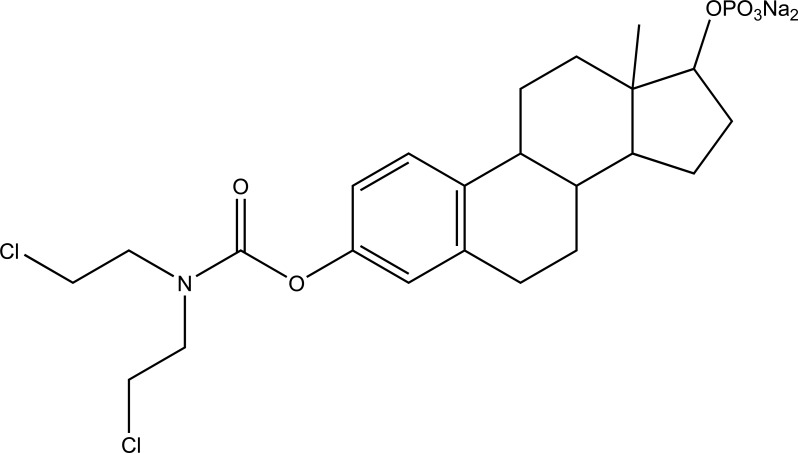
Estramustin.

**Fig. (18):**
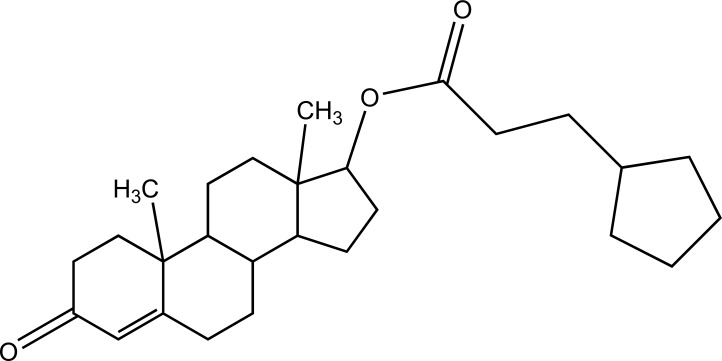
Testosterone cypionate.

**Fig. (19):**
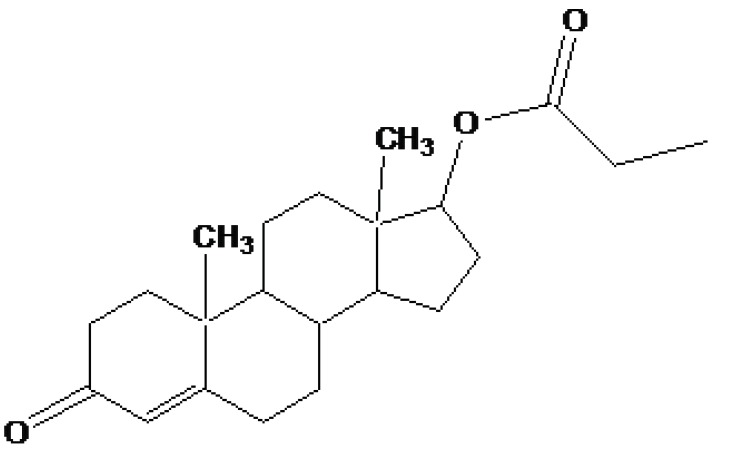
Testosterone propionate.

**Fig. (20):**
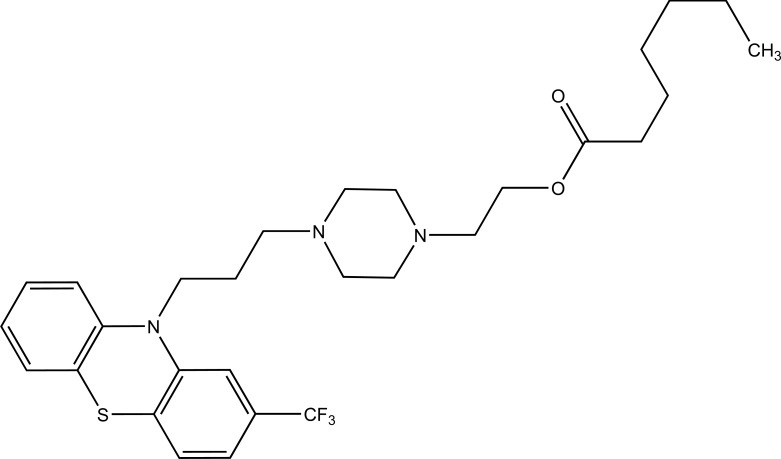
Fluphenazine enanthate.

**Fig. (21):**
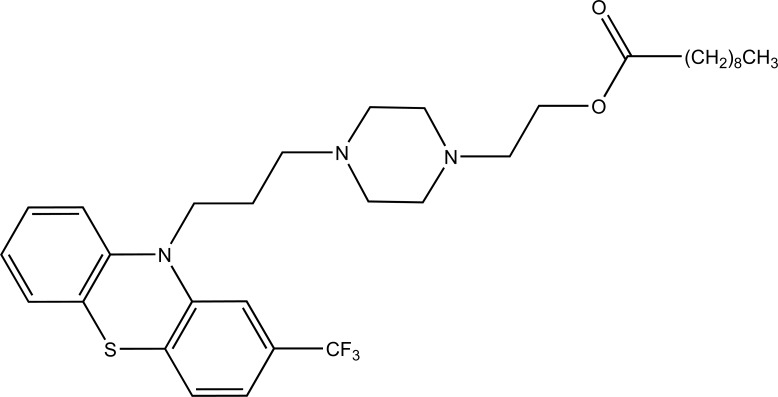
Fluphenazine deaconate.

Taste is one of the important parameters of patient acceptability. The bitter taste of drug can be moderated by prodrug designing [9], for example, chloromphenicol palmitate (Fig. **8**). Odour is another parameter for bitter odour drugs whose boiling points are low. Such liquids generally have a strong odour for *e.g*. Ethyl mercaptan (Fig. **9**) [10]. Several drugs cause gastric irritation for example NSAIDs. This can be overcome by use of prodrug approach *e.g*. Salsalate (Fig. **10**). Some drugs when given by intramuscular injection cause pain. It may be due to weakly acidic nature or poor aqueous solubility. For example, Clindamycin hydrochloride solution and Phenytoin solution cause pain on injection. This can be overcome by formulating their salts i.e. clindamycin phosphate and phenytoin phosphate (Fig. **11**) [11]. The prodrug concept may be utilized to alter the solubility of a drug. This concept is used for eradicating the solubility problem [12]. For example, Ester prodrugs of chloramphenicol have good aqueous solubility, as chloramphenicol succinate and chloramphenicol palmitate (Fig. **12**). However, for the steroidal drugs such as Cortisol (Fig. **13**), its phosphate esters are prepared. Lipophilicity is an important parameter that governs absorption and distribution of drugs. So bioavailability can be improved on altering the lipophilicity, *e.g*. a prodrug of Ampicillin is Pivampicillin (Fig. **14**) showed good absorption. The Glycerol ester of Naproxen (Fig. **15**) produced lesser gastric troubles and had greater plasma concentration. Another prodrug of Naproxen with propyphenazone is synthesised (Fig. **16**) with the view to improve therapeutic index that masks the GI troubles [13]. There are several approaches in drug design to deliver the drug at a specific site. One of the approaches is prodrug concept [14]. For example, Estramustin (Fig. **17**) consists of a phosphorylated steroid, coupled to a normustard through a carbamate linkage. The prodrug concept can be useful for those drugs which have small biological half lives. There are certain examples available in market for example ester-based prodrugs of glucagon-like peptide 1 [15], ester prodrugs of steroids (Testosterone cypionate (Fig. **18**) and propionate (Fig. **19**), Estradiol propionate and Fluphenazine enanthate (Fig. **20**) and deaconate (Fig. **21**) used as depot injections.

## 6 DIFFERENT SYNTHESIZED PRODRUG TO IMPROVE GASTROINTESTINAL TOXICITY

**Fig. (22):**
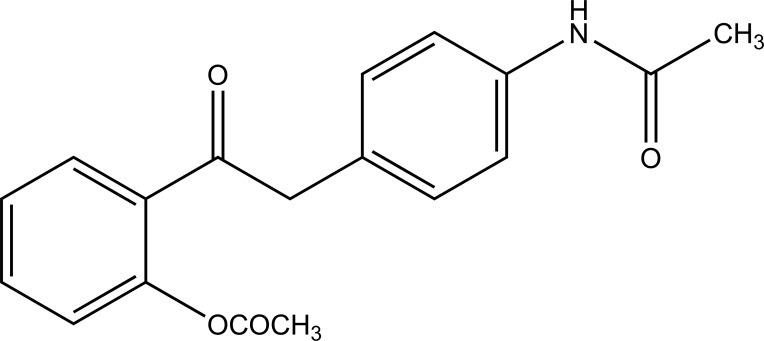
Mutual prodrugs of aspirin and paracetamol.

**Fig. (23):**
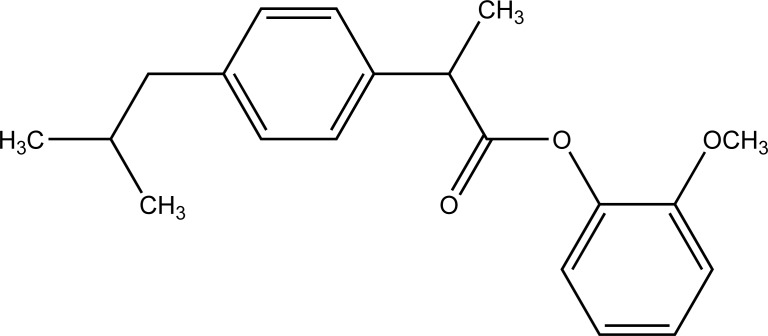
Ibuprofen guiacol ester.

**Fig. (24):**
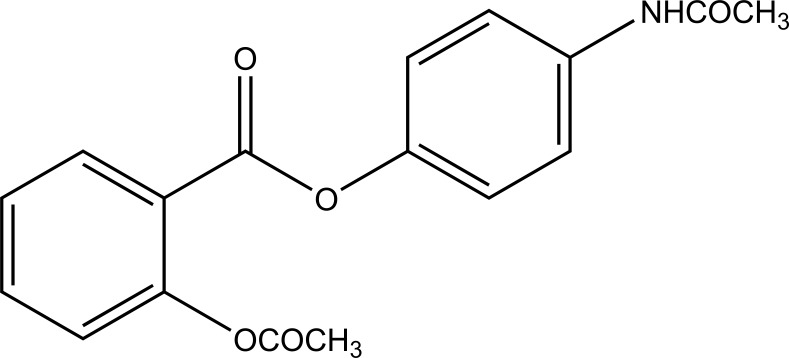
Acetylsalicylic acid and paracetamol prodrug.

**Fig. (25):**
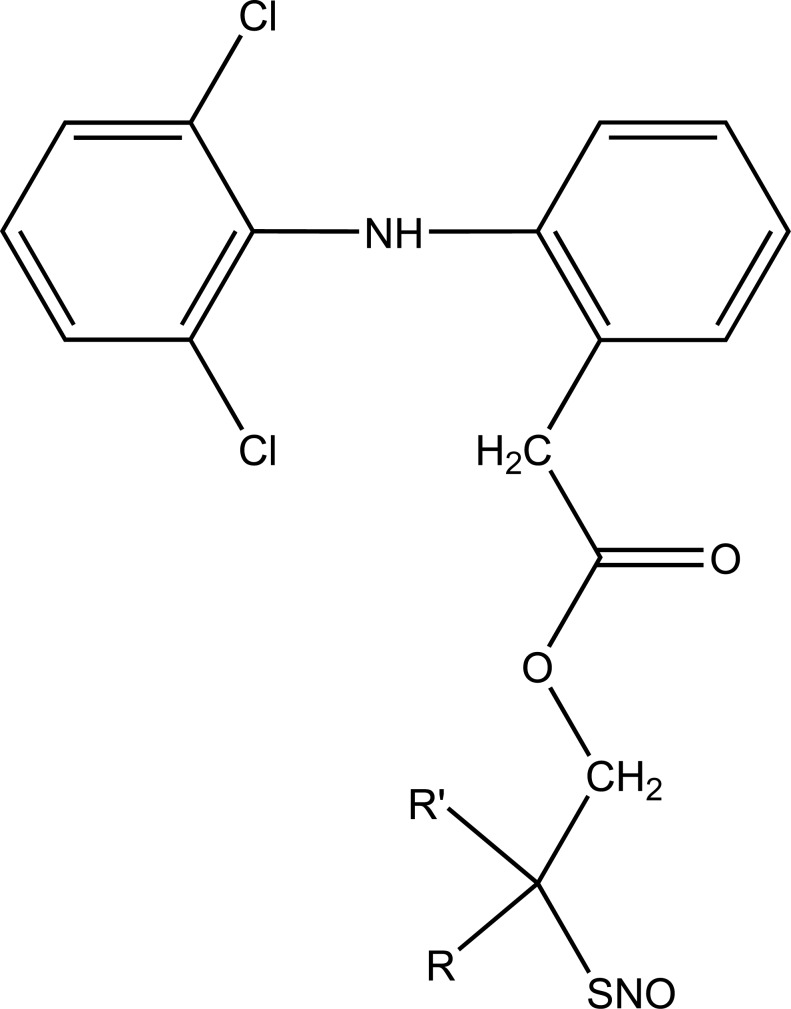
Indomethacin ester prodrug.

**Fig. (27):**
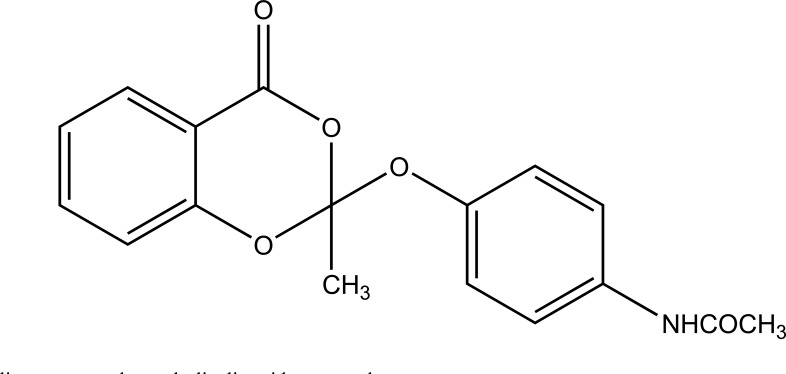
Cyclic paracetamol acetylsalicylic acid ester prodrug.

Despite enormous work carried out on the development of Nobel anti-inflammatory agents, their clinical usefulness is still restricted by their side-effects. The need of safe NSAIDs is still there. As an outcome the search for a prodrug or mutual prodrug with reduced toxicity has continued during recent years. Some significant, reported examples, where prodrug and mutual prodrug concept has been used to overcome GIT side-effects of NSAIDs and other undesirable properties associated with various NSAIDs are complied. A mutual prodrug is synthesized with aspirin and paracetamol (Fig. **22**) linked through ester linkage [16]. The synthesized prodrug abolished the gastrointestinal toxicity. The toxicological and pharmacological profile is evaluated with ibuprofen guiacol ester (Fig. **23**) [17]. The synthesized ester had lesser gastrointestinal toxicity in comparison to pure ibuprofen. The mutual prodrug is synthesized consisting of acetylsalicylic acid and paracetamol (Fig. **24**) [18]. This prodrug did not hydrolyze in the gastric juice and was slowly absorbed than either acetylsalicylic acid or paracetamol. It has been hydrolyzed quantitatively to the parent drugs. Thus there was reduced risk of irritation of gastric mucosa and paracetamol inhibited the erosion action of acetylsalicylic acid by stimulating the stomach prostraglandin synthetase. The prodrug synthesized with a novel indomethacin ester prodrug, 2-[N-[3-(3-(piperidinomethyl) phenoxy) propyl] carbamoylmethylthio] ethyl 1-(p-chlorobenzoyl)-5-methoxy-2-methyl-indole-3-acetate (Fig. **25**). The compound showed antiinflammatory acitivity similar to indomethacin. On the molar basis, the gastric lesioning properties of prodrug was near one hundred times less than indomethacin, result in twenty times improvement in the antiedema activity to ulcerogenicity. It synthesized nitroxybutylesters of flubiprofen (Fig. **26a**) and ketpprofen (Fig. **26b**) and evaluated them for anti-inflammatory activity and gastrotoxicity [19]. A cyclic paracetamol acetylsalicylic acid ester has been reported (Fig. **27**). This has been claimed to undergo enzymatic hydrolysis to release parent drugs, acetylsalicylic acid and paracetamol [20].

## 7 RESULTS AND DISCUSSION

**Fig. (28):**
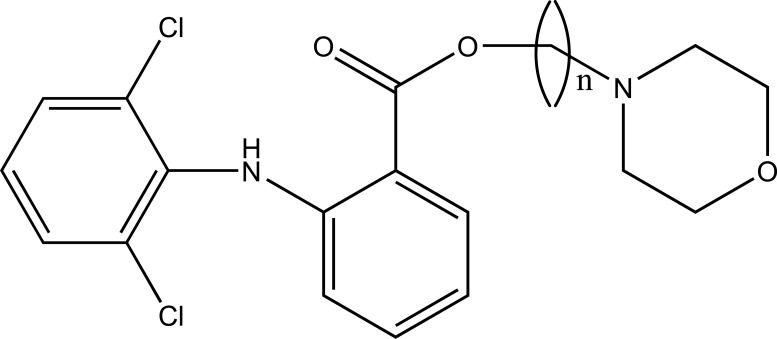
Ester prodrugs of diclofenac.

The prodrug is synthesized with morpholinoalkyl ester prodrugs of diclofenac for oral delivery (Fig. **28**) and evaluated for *in vitro* and *in vivo* analysis for their potential use as prodrugs [21]. It was screened for hydrolytic activity in SGF/pH 7.4 phosphate buffer and rat plasma, respectively, at 37 °C. They were significantly less irritating to gastric mucosa than parent drug administration by oral routes to rats.

**Fig. (29):**
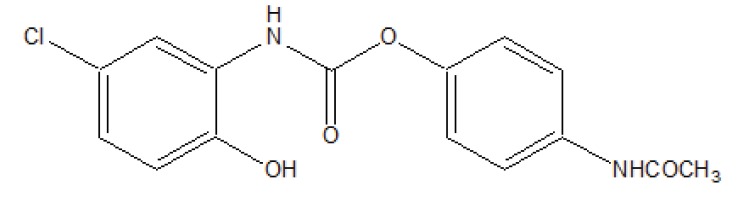
Mutual prodrug of chloroxazone and acetaminophen.

**Fig. (30):**
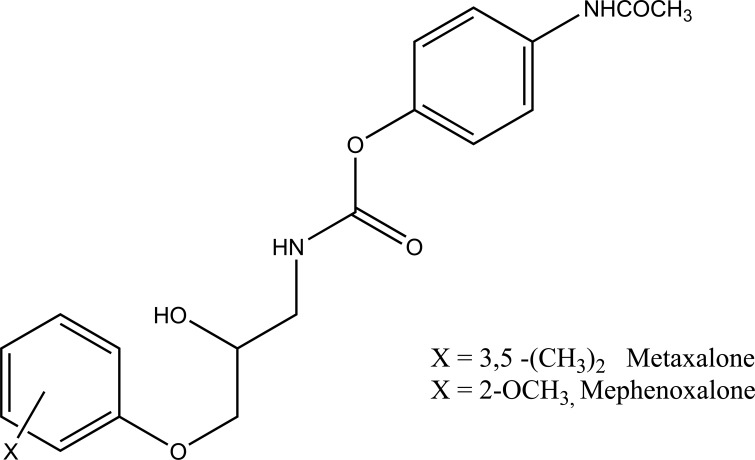
Carbamate codrugs.

The prodrug is synthesized with the series of alkyl and aryl N-(5-chloro-2-hydroxyphenyl) carbamates. The prodrugs of chlorzoxazone and acetoaminophen are shown in figure [22] (Fig. **29**). The carbamate co-drugs (Fig. **30**) that quantitatively releases acetaminophen and the corresponding active oxazolidinones-metaxalone and mephenoxalone respectively [23].

**Fig. (31):**
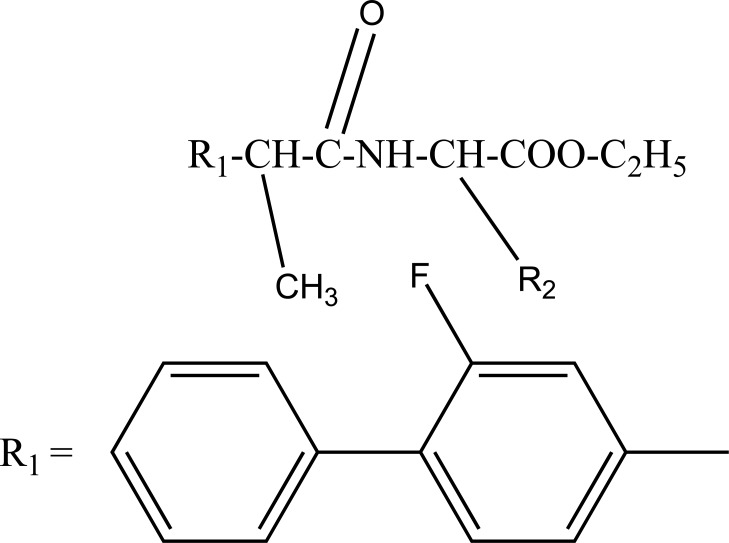
Ethyl esters of flurbiprofen with arginine, lysine and p-guanidine L- phenylananine.

The prodrug prepared with ethyl esters (Fig. **31**) of flurbiprofen with arginine, lysine and *p*-guanidine L- phenylananine and evaluations are carried out for its kinetics of hydrolysis [24]. The prodrug prepared with hydroxyethyl esters of mefenamic acid (Fig. **32a**) and diclofenac are illustrated in figure (Fig. **32b**) [25]. Its hydrolytic studies were done in aqueous buffer solutions and also in human plasma. The result showed that the degradation of diclofenac ester in aqueous buffer solutions was less as compared to hydrolysis in plasma. While the mefenamic acid ester showed greater t_1/2_ in buffer solutions as well as in the plasma.

**Fig. (33):**
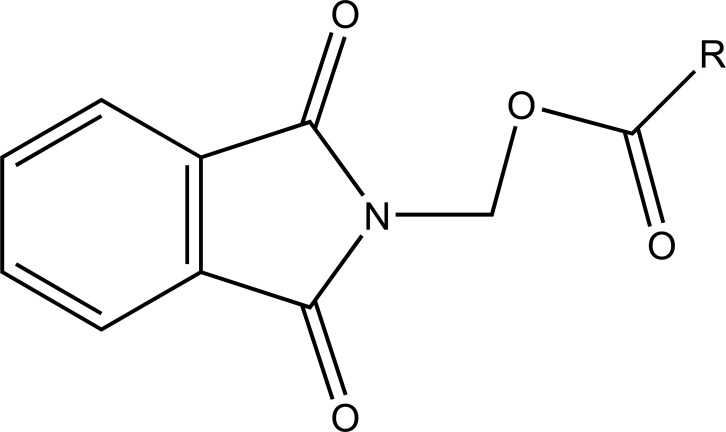
N- Hydroxymetyl phthalimide esters.

**Fig. (34):**
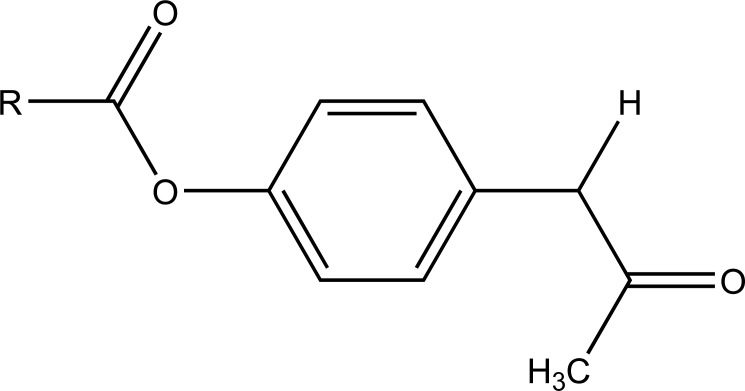
Prodrug of paracetamol esters.

The carboxylic acid function was masked of some NSAIDs via synthesis of *N*- Hydroxymetyl phthalimide esters (Fig. **33**). These were found to be potential prodrugs [26]. The prodrug is synthesized with paracetamol esters (Fig. **34**) of some NSAIDs (aspirin, ibuprofen, naproxen, diclofenac, flufenamic acid and indomethacin) and evaluated for gastric toxicity [27]. The synthesized prodrugs had a better therapeutic index than the parent drugs [28].

**Fig. (35):**
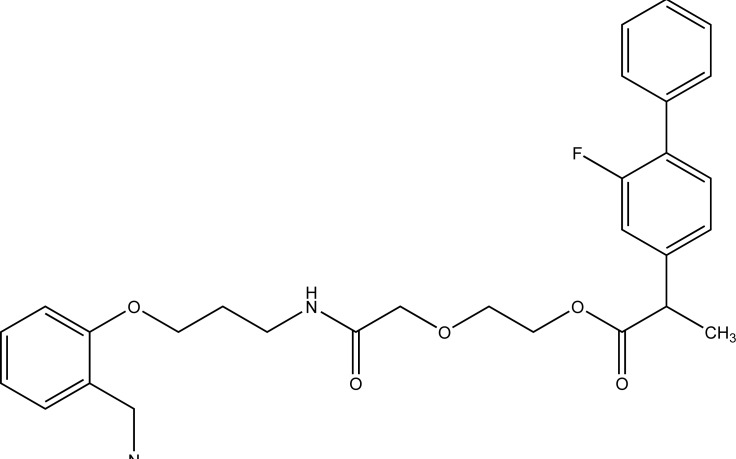
Conjugate of Flubiprofen with histamine.

**Fig. (36):**
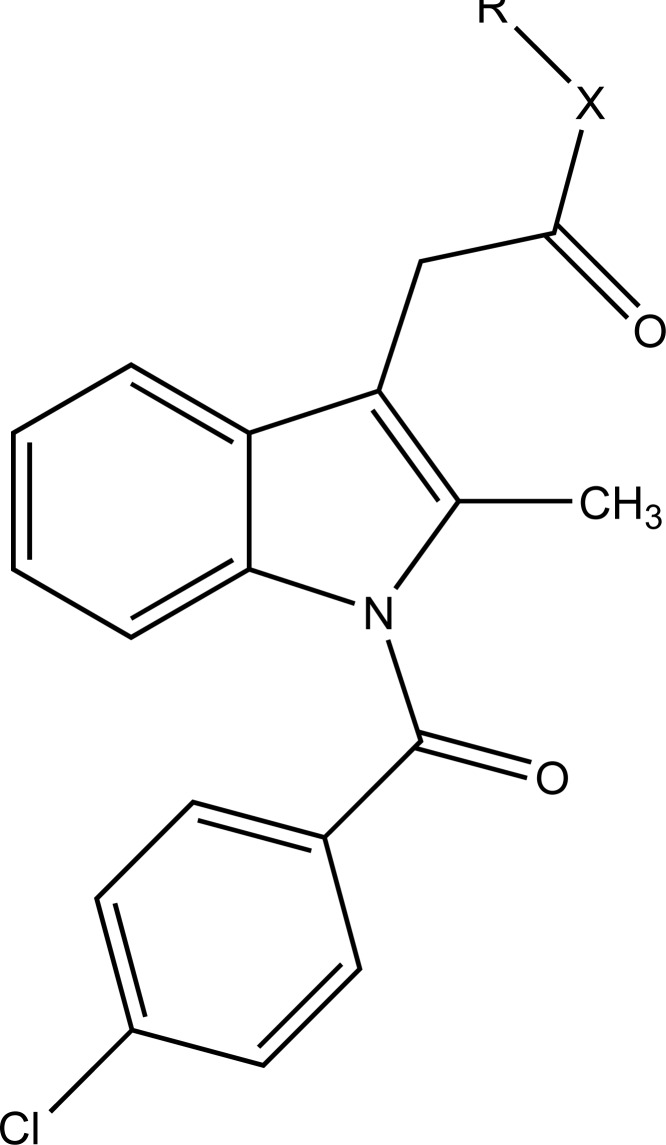
Prodrug of indomethacin and meclofenamic acid.

The Flubiprofen was conjugated with histamine H_2_ antagonists (Fig. **35**) and screened for the reduction in gastric damage by NSAID, and examined for pharmacological properties. Indomethacin and meclofenamic acid were derivatized with 5,8,11,14-eicosatetraynoic acid (Fig. **36**). The synthesized prodrugs act as potent and selective cyclooxygenase-2 (COX-2) inhibitors [29].

**Fig. (37):**
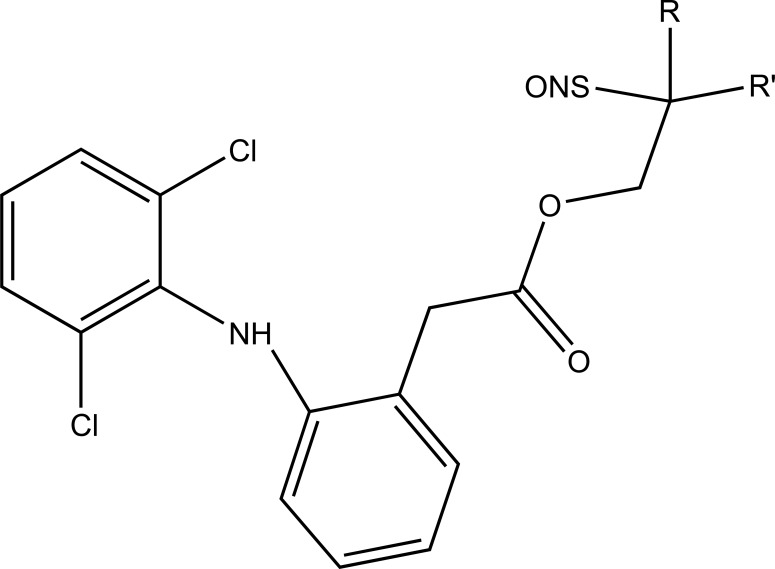
Nitrosothiol esters of diclofenac.

The novel diclofenac esters containing a nitrosothiol (−S-NO) prodrug were synthesized (Fig. **37**). The synthesized drugs evaluated *in vivo* for bioavailability, pharmacological activity, and gastric irritation [30]. The synthesized S-NO-diclofenac derivatives elicited comparable activity to those of diclofenac in the carrageenan-induced paw edema test and phenylbenzoquinone-induced writhing test, respectively.

**Fig. (38):**
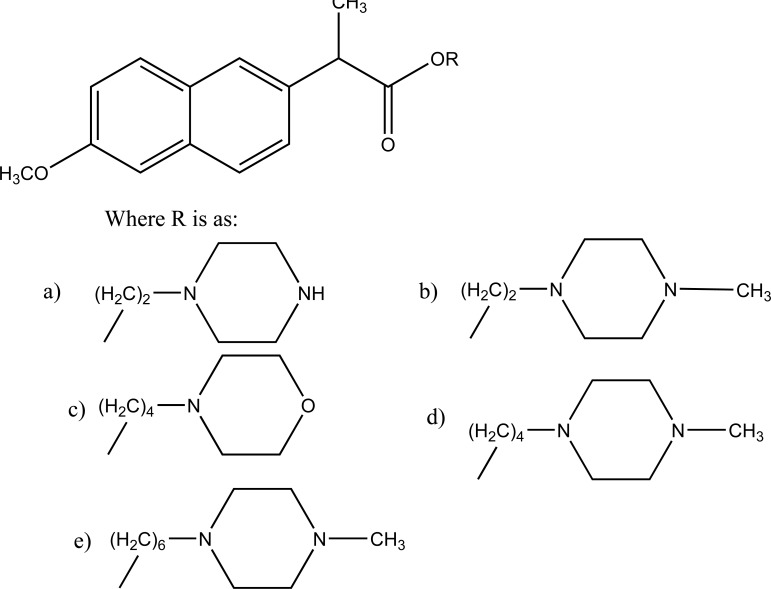
Morpholinyl and piperazinylalkyl esters of naproxen.

The prodrugs of naproxen having morpholinyl and piperazinylalkyl structures were synthesized (Fig. **38**). These prodrugs were administered topically that showed [31] 4 to 9 fold enhancement of permeation for 38a and 38b and a 4 four-fold better permeation for 17b.

The prodrug of aspirin and nitric oxide was prepared with derivatives of isosorbide-5-mononitrate (ISMN) (Fig. **39a** and **39b**) and evaluated for hydrolytic activity [32]. The ester derivative of ISMN showed prominent hydrolysis in plasma solution than in buffer solution.

**Fig. (40):**
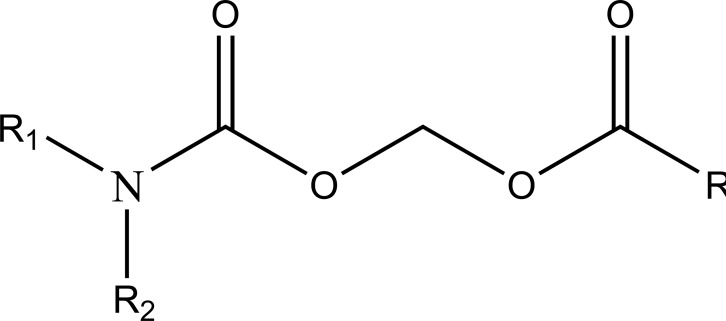
Aminocarbonyloxymethyl esters.

The prodrugs of naproxen and flufenamic acid were synthesised and evaluated for lipophilicity and their hydrolysis was done in aqueous solutions and human plasma (Fig. **40**) [33].

**Fig. (41):**
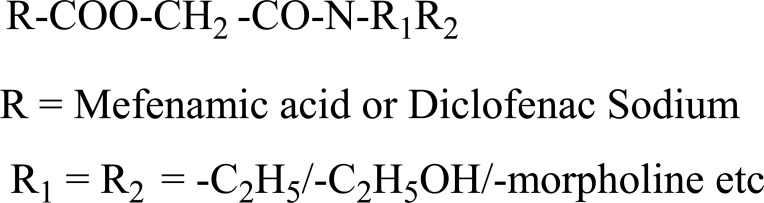
Glycosamide esters of diclofenac and mefenamic acid.

**Fig. (42):**
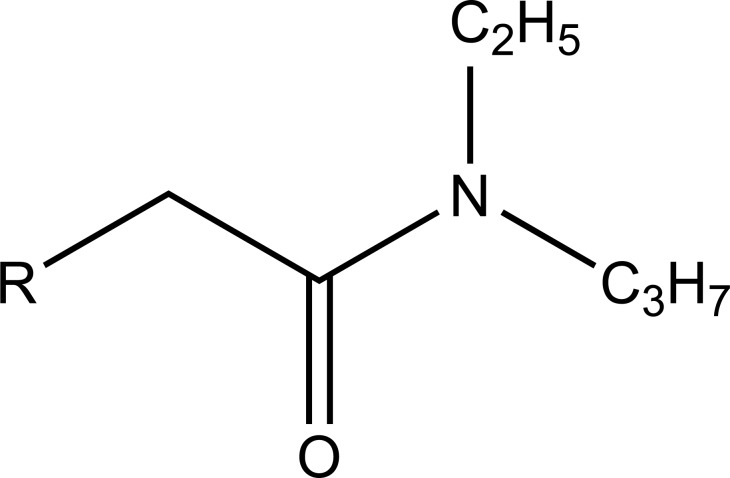
Glycolamide ester prodrugs.

The prodrug of diclofenac and mefenamic acid was synthesized and evaluated (Fig. **41**) [34]. The glycolamide ester prodrugs of ibuprofen, diclofenac, naproxen and indomethacin (Fig. **42**) were prepared and studied for their GI toxicity in rats and studied their different physicochemical, pharmacological and toxicological parameters were studied [35].

The prodrugs of ketoprofen (Fig. **43a**), naproxen (Fig. **43b**) and diclofenac (Fig. **43c**) were synthesized as polyoxyethylene esters and showed better stability in phosphate buffer (pH 7.4) and simulated gastric fluid (pH 2.0) [36] while easily hydrolyzed by human plasma. The pharmacological activities such as, anti-inflammatory and analgesic activities of prodrugs were found to be same to as the parent drugs; while at higher doses, prodrugs were shown to have reduced gastric irritation.

**Fig. (45):**
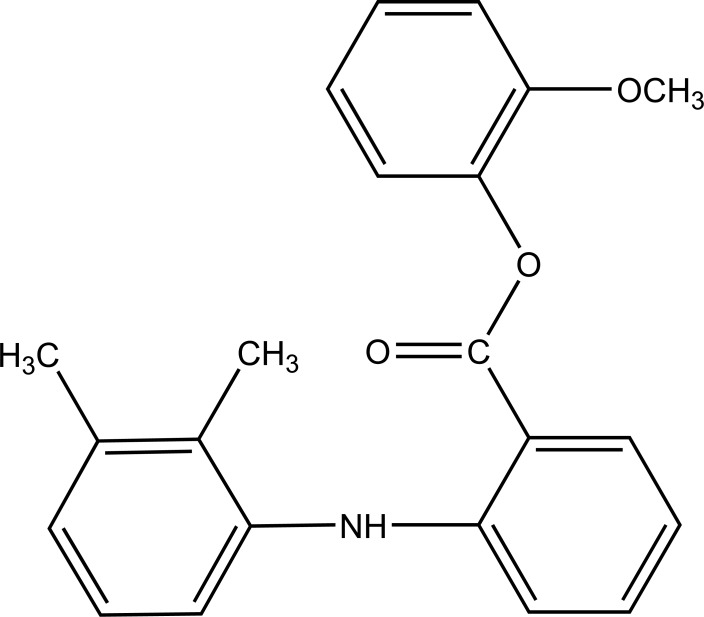
Mefenamic acid-guaiacol ester.

The prodrug of meclofenamic acid as amides was synthesized as selective cyclooxygenase-2 inhibitors (Fig. **44a** and **b**) [37]. Mefenamic acid-guaiacol ester (Fig. **45**) as prodrug was synthesized and investigated for its physicochemical properties, stability and transport across Caco-2 monolayers [38].

**Fig. (46):**
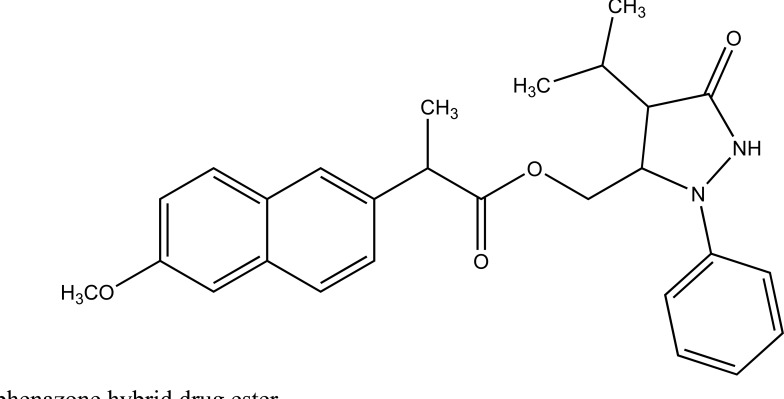
Naproxen-propyphenazone hybrid drug ester.

**Fig. (47):**
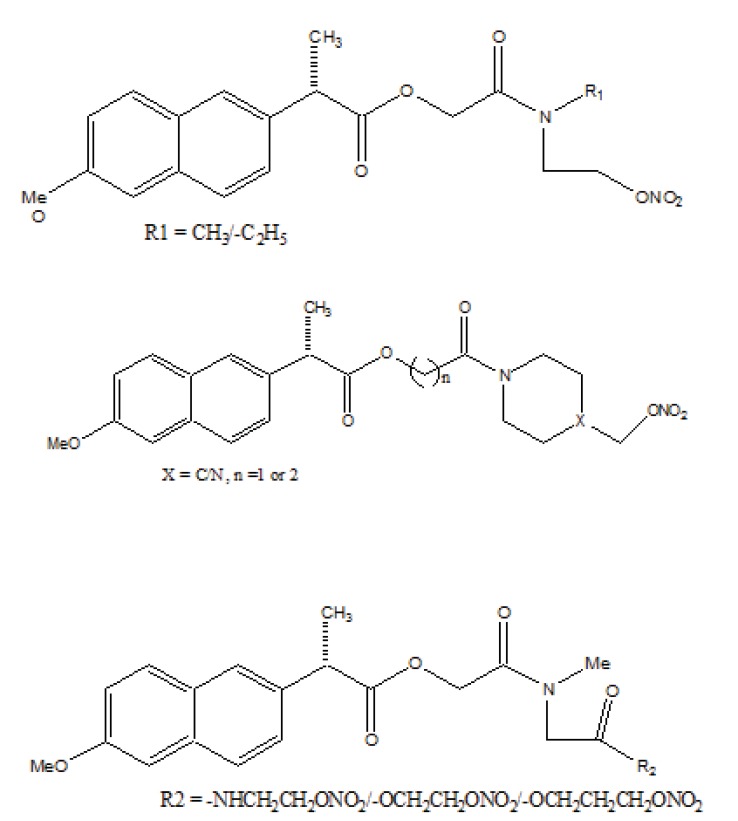
Glycolamide naproxen prodrugs.

Naproxen–propyphenazone hybrid drug ester (Fig. **46**) and/or amide synthesized and showed improvement in the therapeutic index of the parent drug [39]. A series of glycolamide naproxen prodrugs (Fig. **47**) bearing a nitrate group as a nitric oxide (NO) donor group has been synthesised [40]. Their anti-inflammatory activity, naproxen release, and gastric tolerance were evaluated. These NO-donor glycolamides were found to be safer NSAIDs with similar activity.

**Fig. (48):**
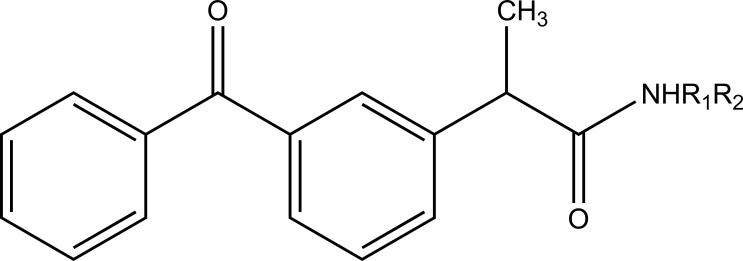
Ketoprofenamides.

A series of new ketoprofen amides as potential NSAID prodrugs has been described (Fig. **48**) [41]. The ketoprofen benzotriazolide treated with various amines (primary, secondary, hydroxylamine and amino acid β-alanine) to form amide.

**Fig. (49):**
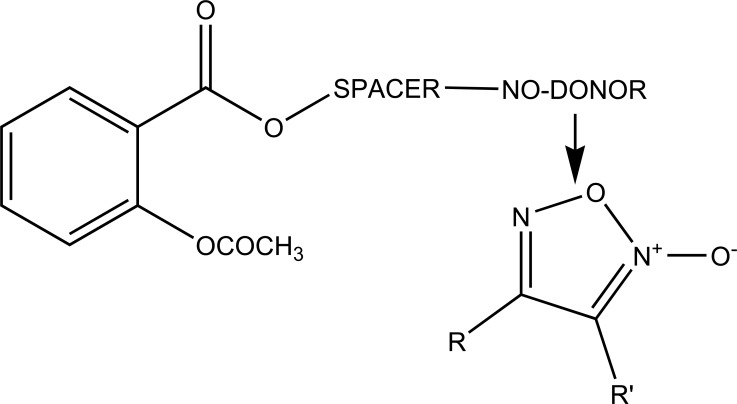
NSAIDs prodrugs.

The aspirin is coupled with ester linkage to furoxan moieties to give a new series of NSAIDs (Fig. **49**) and evaluated for NO-releasing, pharmacological and ulcerogenic properties [42]. The references taken in this experiment were furazan derivatives, its propyl ester, and its γ-nitrooxypropyl ester. All the synthesized prodrugs showed anti-inflammatory activity and spare the acute gastrotoxicity and showed an antiplatelet activity that may be due to release of NO.

**Fig. (50):**
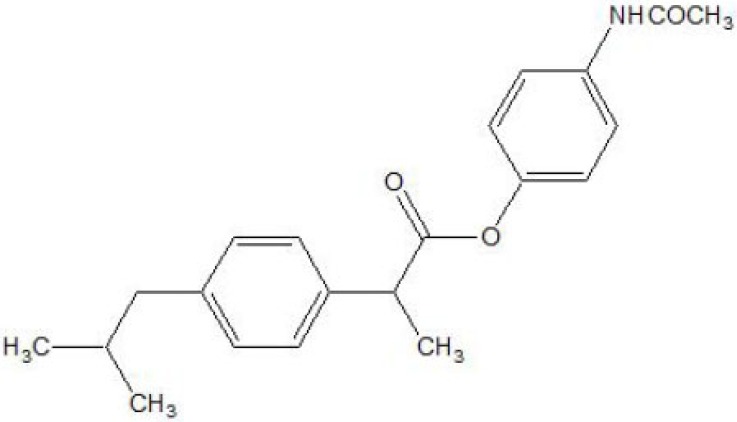
Ibuprofen with paracetamol.

**Fig. (51):**
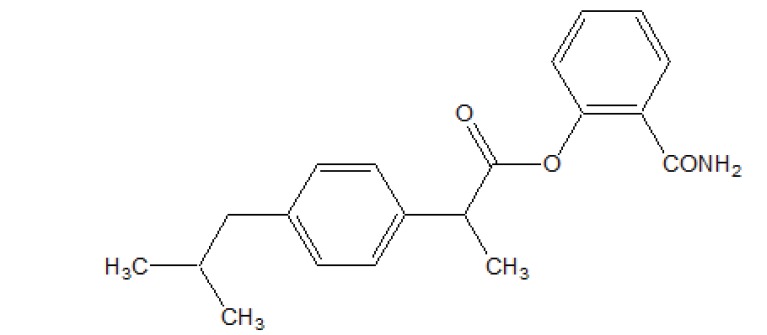
Ibuprofen with salicylamide.

**Fig. (52):**
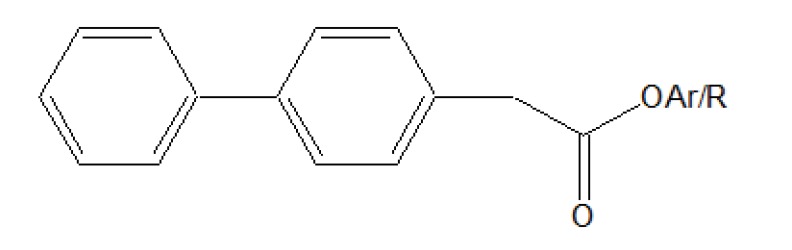
Mutual prodrugs of 4-BPA.

The prodrugs of ibuprofen with paracetamol (Fig. **50**) and salicylamide (Fig. **51**) were synthesized and reported better lipophilicity and reduced gastric irritation [43]. The mutual prodrugs (Fig. **52**) of 4-BPA with naturally occurring phenolic antioxidants like thymol, guaiacol, eugenol, and other alcoholic compounds were synthesized [44]. The 4-BPA prodrugs showed better gastro sparing activity.

**Fig. (53):**
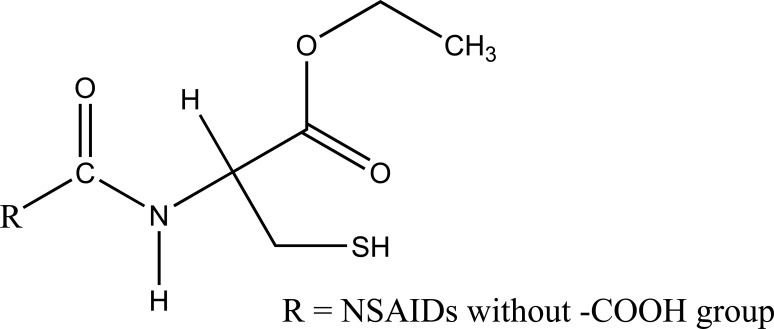
NSAIDs with L-cysteine ethyl ester.

A series of amide derivatives of NSAIDs with L-cysteine ethyl ester were synthesized and evaluated (Fig. **53**) [45]. The synthesized prodrugs showed reduced gastrointestinal toxicity with retention of anti-inflammatory, antioxidant and hypocholesterolemic–hypolipidemic activity. This structural design provided a path to develop a safer anti-inflammatory agent, which can be used in conditions such as neurodegenerative disorders.

**Fig. (54):**
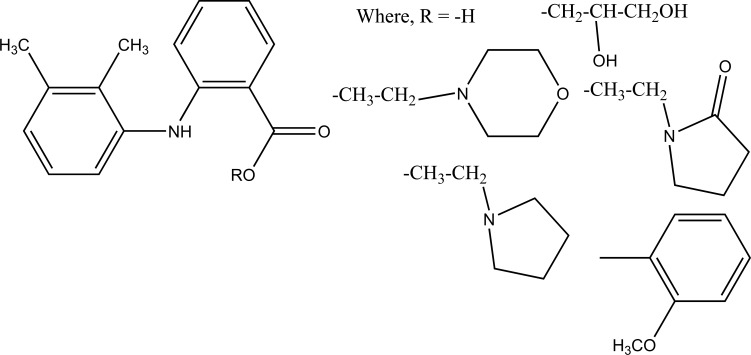
Ester derivatives of mefenamic acid.

The prodrugs of mefenamic acid as ester derivatives were synthesized (Fig. **54**). The synthesized prodrug checked for enzymatic stability and bidirectional permeability across Caco-2 monolayer. This series was made with aim of suppressing local gastrointestinal toxicity [46].

**Fig. (55):**
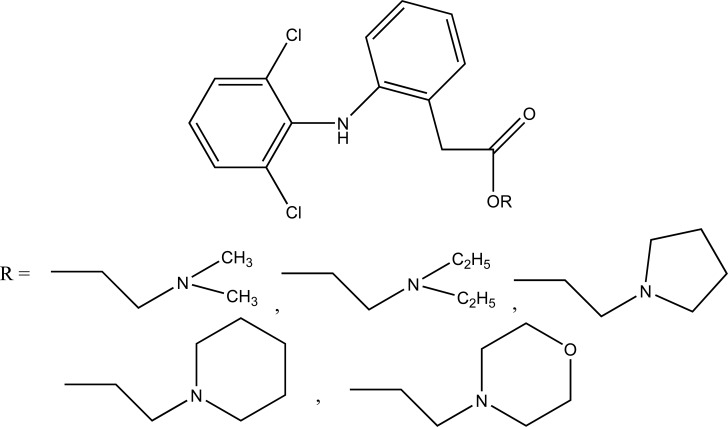
N,N- disubstituted aminoethyl ester derivatives of diclofenac.

The N,N- disubstituted aminoethyl ester derivatives of diclofenac were synthesized and evaluated five different esters (Fig. **55**) [47]. These esters were designed by blocking the acidic carboxyl group. It was designed in such a way that it possesses the anticholinergic activity in intact form before cleavage. The synthesized prodrugs reduce gastric toxicity and thereby lessen the local irritation.

**Fig. (56):**
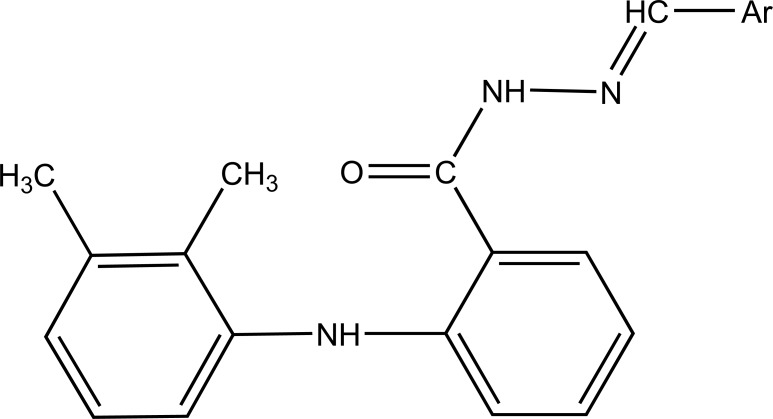
N- arylhydrazone derivatives of mefenamic acid.

The *N*- arylhydrazone derivatives of mefenamic acid were synthesized (Fig. **56**) and evaluated for analgesic and anti-inflammatory activity [48].

**Fig. (57):**
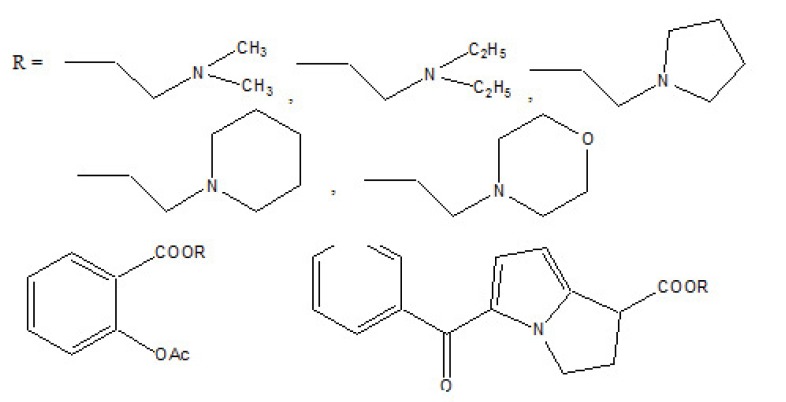
N,N-disubstituted aminoethyl ester derivatives of aspirin and ketorolac.

**Fig. (58):**
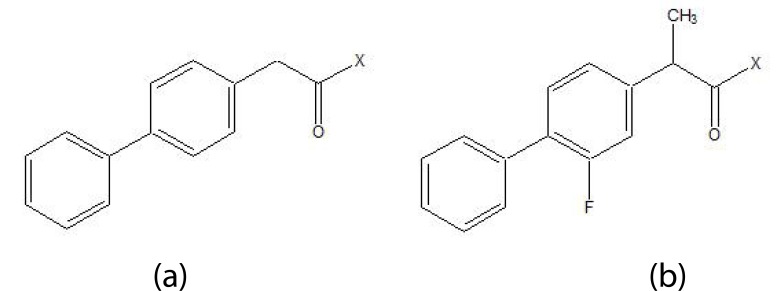
N,N-disubstituted amino-ethyl ester derivatives.

The five different *N,N*-disubstituted aminoethyl ester (Fig. **57a** and **b**) derivatives of aspirin and ketorolac [49] were synthesized and evaluated for hydrolytic stability at different buffer solution and in serum. The data showed that it had fast hydrolysis at human plasma with retention of gastric toxicity. The ulcerogenic potential of the evaluated derivatives was significantly reduced. However, the anti-inflammatory activity in case of aspirin derivatives was much lower. The different *N*, *N*- disubstituted amino-ethyl ester derivatives containing [1,1′-biphenyl]-4-acetic acid and flurbiprofen (Fig. **58a**; **b**) were synthesized. All the prodrugs were screened for hydrolytic stability and found significant result. The compounds were found to possess less ulcerogenic potency with slight higher anti-inflammatory activity.

**Fig. (59):**
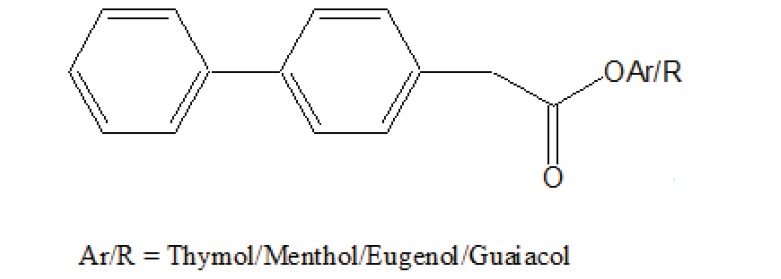
l-proline, trans-4-hydroxy-l-proline or dl pipecolinic acid prodrugs.

The prodrug of NSAID (ibuprofen/indomethacin) and an antioxidant moiety was made through amide bonds to form structures like *l*-proline, *trans*-4-hydroxy-*l*-proline or *dl* pipecolinic acid (Fig. **59**) [50]. The synthesized compounds were found to possess reduced gastrointestinal problems. The prodrugs were found to retain anti-inflammatory and antioxidant activities.

**Fig. (60):**
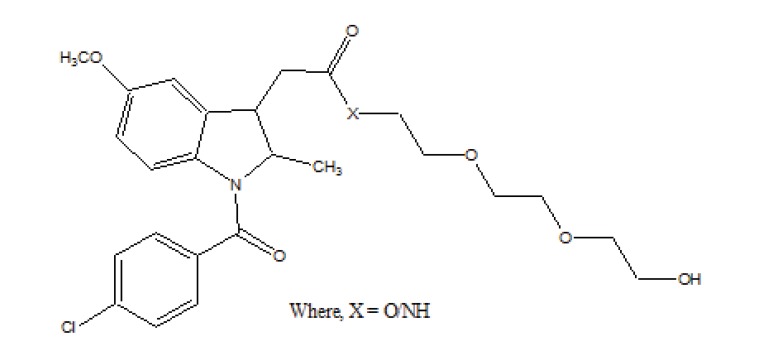
Indomethacin-TEG (Triethylene Glycol) ester and amide prodrugs.

The prodrug is synthesized and studied the *in vitro* enzymatic and non-enzymatic hydrolysis of indomethacin–TEG (Triethylene Glycol) ester and amide prodrugs (Fig. **60**) [51]. It was found that the ester conjugates were stable at pH 3 and 6 while showed greater hydrolysis in buffered plasma. The amide conjugate formed was found to be stable.

**Fig. (61):**
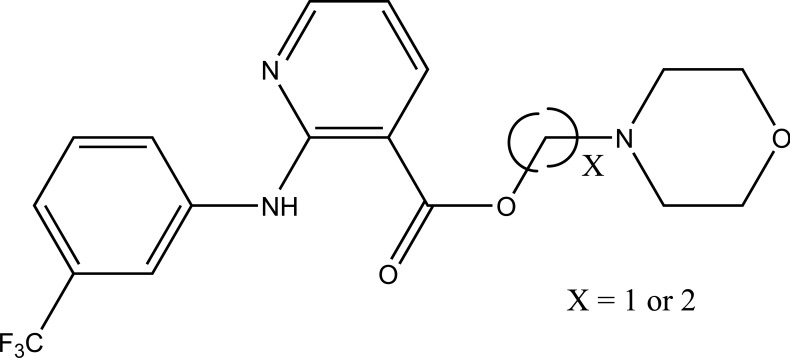
Morpholinoalkyl ester prodrugs of niflumic acid.

The prodrug is prepared with novel morpholinoalkyl ester prodrugs (Fig. **61**) of niflumic acid by esterification of appropriate morpholinylalkyl alcohols [52]. The ester prodrugs were quantitatively hydrolyzed to the parent drug niflumic acid by enzymatic and/or chemical means. The observation of the experiment results in an increase in the carbon chain length making the prodrugs more stable in phosphate buffer (pH 7.4) than in pH 1.3 (SGF), but they were rapidly hydrolyzed in human plasma. The synthesized compounds showed good anti-inflammatory activity and also screened for *in vivo* ulcerogenicity. The prodrugs were significantly less irritating to gastric mucosa.

**Fig. (62):**
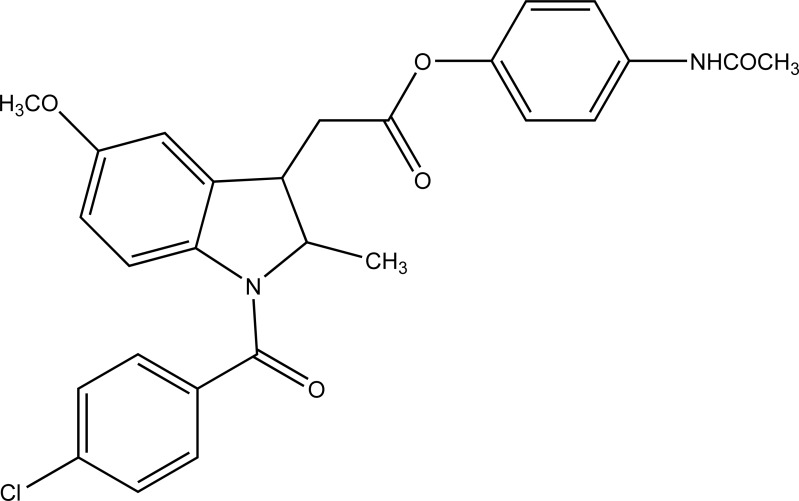
Indomethacin with paracetamol mutual prodrug.

The prodrug of indomethacin with paracetamol was prepared as mutual prodrug (Fig. **62**) [53]. The mutual prodrugs were devoid of gastric irritation. The reason may be due to its unionized form in acidic pH and ionized form in alkaline pH. That is why it is not absorbed in stomach.

**Fig. (63):**
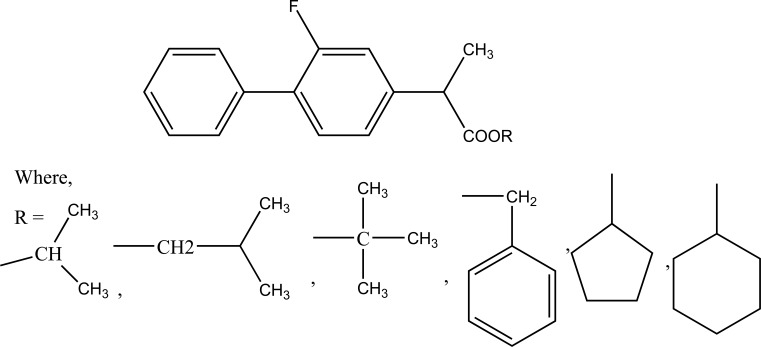
Alkyl ester prodrugs.

The nine alkyl ester prodrugs (Fig. **63**) of flurbiprofen synthesized with an aim to reduce its gastrointestinal side-effects [54]. These were subjected to plasma hydrolysis and gastrointestinal toxicity studies. The plasma hydrolysis studies indicated that methyl and propyl prodrugs of flurbiprofen undergo faster hydrolysis as compared to the remaining ester prodrugs. The ulcer index study showed that n-propyl, iso-propyl, benzyl and cyclopentyl prodrugs of flurbiprofen were less irritating to the gastric mucosa as compared to the parent drug.

**Fig. (64):**
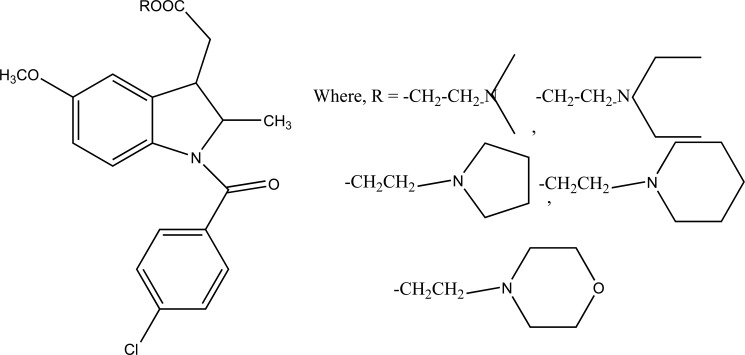
N,N-disubstituted aminoethanol ester.

The prodrug of indomethacin bearing same structure to the aminoethanol ester class of anticholinergics was synthesized and evaluated (Fig. **64**) [55] for gastric toxicity. All the synthesized drugs were less irritating to the gastric mucosa than the parent drug. The pharmacological studies indicated that the synthesized compounds have gastrosparing potential.

**Fig. (65):**
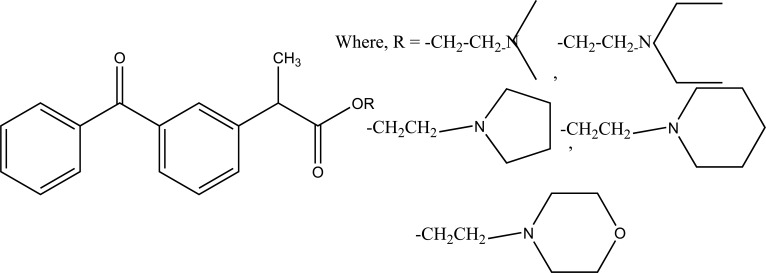
N,N-disubstituted aminoalcohol ester.

The *N,N-*disubstituted aminoalcohol ester derivatives (Fig. **65**) of ibuprofen and ketoprofen [56] were synthesized. All the esters were experimentally found to have proven good antiinflamatory and anticholinergic activities. There was significant reduction of ulcerogenicity in the stomach.

**Fig. (66):**
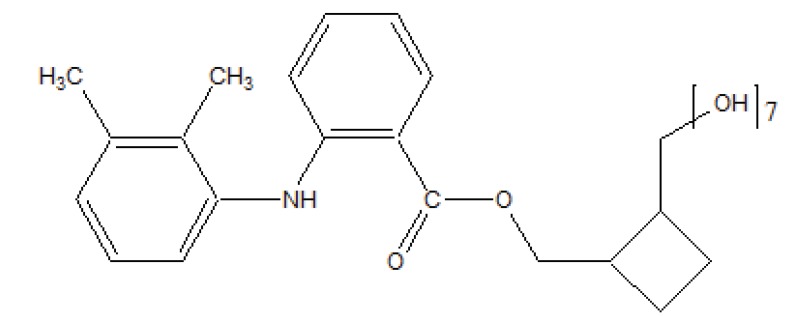
Mefenamic acid prodrug of beta-cyclodextrins.

The prodrug of mefenamic acid with β-cyclodextrins was synthesized (Fig. **66**). β-cyclodextrins have primary hydroxyl group that was used to conjugate the acid group [57] of parent drug. The prodrug formed was evaluated for stability in simulated gastric and intestinal fluid. The hydrolysis kinetics studies of cyclodextrin conjugate in colon were confirmed. The ester formed showed less ulcerogenicity.

**Fig. (67):**
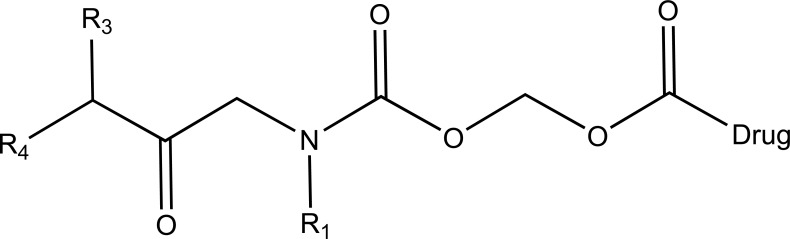
Aminocarbonyloxymethyl esters of diclofenac and flufenamic acid.

The novel aminocarbonyloxymethyl esters (Fig. **67**) of diclofenac and flufenamic acid were synthesized that bear amino acid amide carriers [58]. In non-enzymatic and enzymatic conditions the amino acids prodrugs got hydrolyzed to the parent drug. The use of amino acid carriers with this concept increased the aqueous solubility. So it gave an idea to increase the bioavailability.

**Fig. (68):**
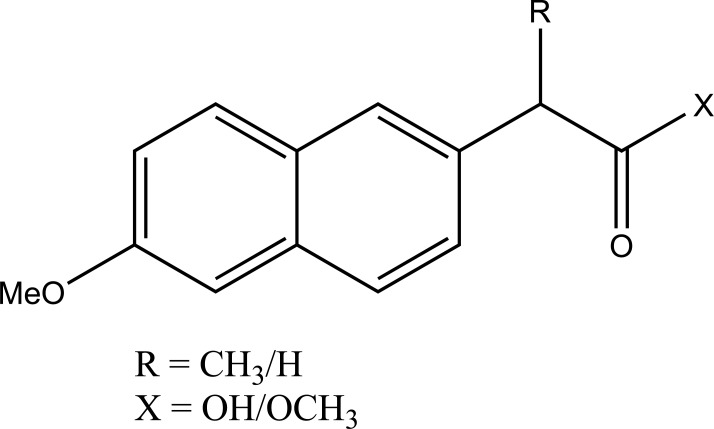
Naproxen and 6-methoxy-2-napthylacetic acid with aminoalcohol ester.

The prodrug of naproxen and 6-methoxy-2-napthylacetic acid with aminoalcohol ester was synthesized. The aminoalcohol ester was a class of anticholinergics (Fig. **68**). On screening of the prodrugs it was found that the derivatives were found to possess good anticholinergic activity with retention of anti inflammatory potency of the parent drug with significant reduction of ulcerogenicity [59].

**Fig. (69):**
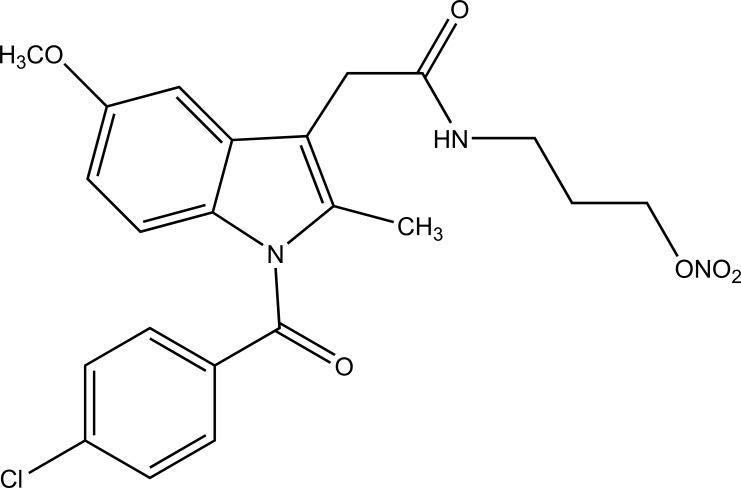
Indomethacin amide-nitrate derivative.

The 3-acetic acid of the indomethacin used to synthesize an amide-nitrate derivative. It was synthesized with the aim to increase selectivity against cyclooxygenase-2 and to increase drug safety by covalent attachment of an organic nitrate moiety as a nitric oxide donor. (Fig. **69**) and a sulfonamide-nitrate derivative elicited COX-2 selectivity [60].

**Fig. (70):**
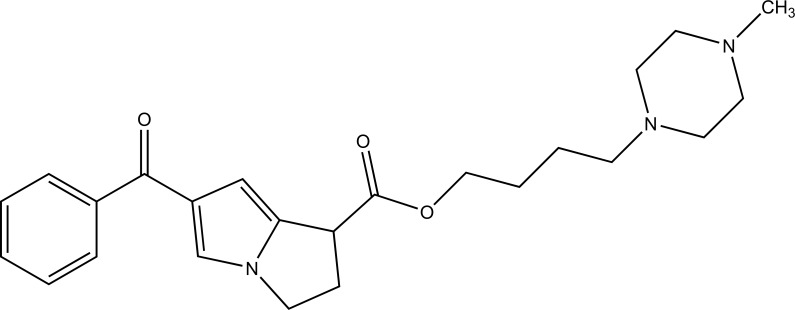
Piperazinylalkyl ester prodrugs of ketorolac.

The prodrug of ketorolac was synthesized with seven piperazinylalkyl prodrugs that increase its skin permeation [61]. The hydrolytic study was carried out in aqueous buffer and in plasma that showed its stability in aqueous buffer while showed prominent release in human plasma. Out of seven prodrugs, one of them (Fig. **70**) showed increased permeation at pH 5 and 7.4. This cleared that it was highly lipophilic at pH 7.4 and better aqueous solubility at pH 5 compared to parent drug.

**Fig. (71):**
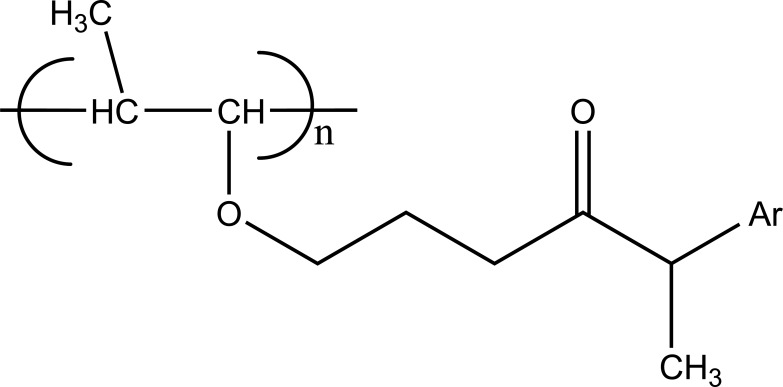
Polymeric prodrugs of ibuprofen, ketoprofen and naproxen.

The prodrugs of ibuprofen, ketoprofen and naproxen, (Fig. **71**) were formed by reacting chloroacylated drugs with amine groups of polymer [62]. They have amide linkage. The drugs got released by rupturing of amide bond. The study clearly indicated that the vinyl ether type polymer was the useful carrier to release of profens in controlled release systems. The release of prodrugs was pH dependent.

**Fig. (72):**
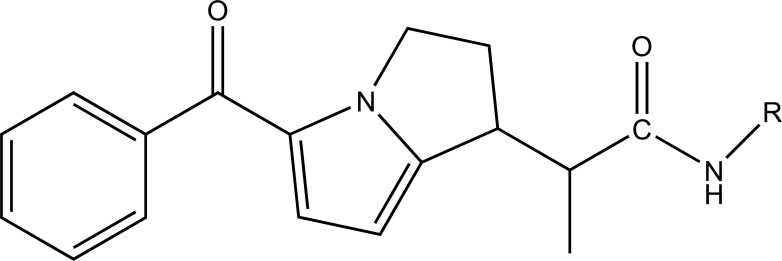
Prodrugs of ketorolac by amidation.

The ten prodrugs of ketorolac were synthesized by reaction with ethyl esters of amino acids. The amino acids selected were glycine, L-phenylalanine, L-tryptophan, L-valine, L-isoleucine, L-alanine, L-leucine, L-glutamic acid, L-aspartic acid and β-alanine (Fig. **72**) [63]. The prodrugs were screened for pharmacological studies and ulcerogenic studies. The result indicated marked reduction of ulcerogenicity and showed comparable analgesic, and anti-inflammatory activities.

**Fig. (73):**
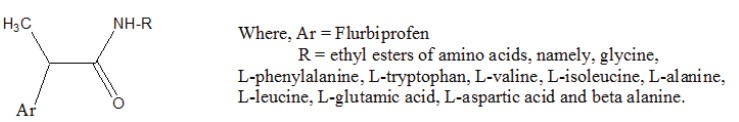
Prodrugs of flubiprofen by amidation.

Similarly the ten prodrugs of flubiprofen were synthesized with same amino acid series (Fig. **73**) [64]. The fruitful results were obtained as in case of ketorolac is obtained.

**Fig. (75):**
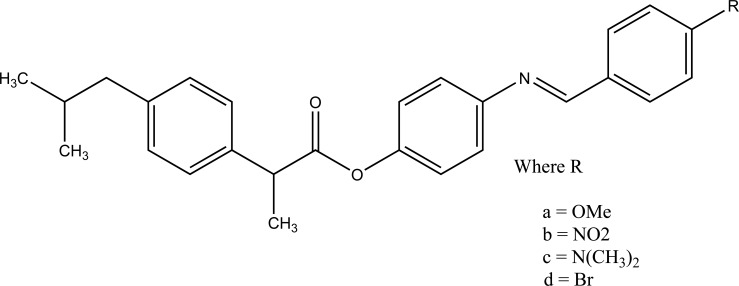
NO-Aspirin prodrug.

**Fig. (76):**
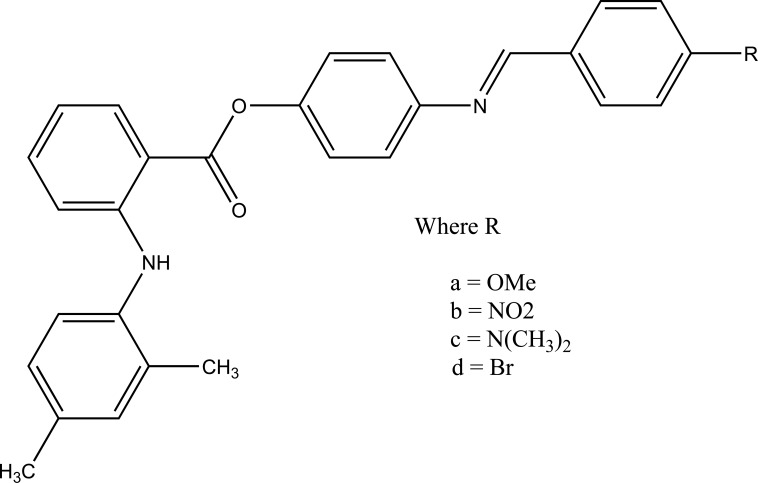
NO-Diclofenac prodrug.

The prodrug of the anti-inflammatory drugs aspirin and indomethacin was made with 1-(2-carboxypyrrolidin-1-yl) diazen-1-ium-1,2-diolate ion via a one-carbon methylene spacer to obtain two new hybrid prodrugs [65]. The aspirin and indomethacin prodrugs were found to be potent one. The ulcerogenic data clearly indicate that prodrug formed was safer one. (Fig. **74a** and **b**). The prodrugs such as NO-Aspirin (Fig. **75**) and NO-Diclofenac (Fig. **76**) were designed, synthesized and evaluated [66]. Although the amide-containing compounds 1d did not show significant bioavailability, the left compounds showed better pharmacokinetic, pharmacological and gastric-sparing properties. However, the NO-Diclofenac had elicited better pharmacological activity and NO-releasing properties.

**Fig. (77):**
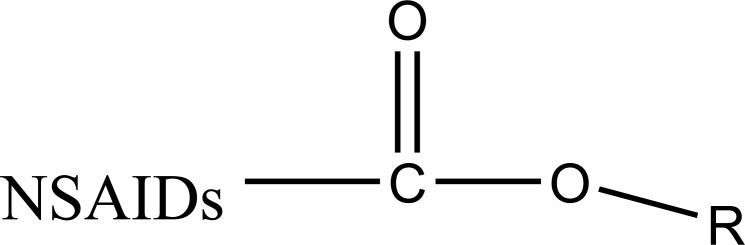
Ester prodrugs of flurbiprofen, ibuprofen and ketoprofen.

**Fig. (78):**
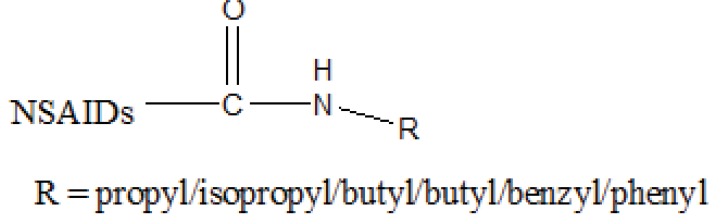
Amide prodrugs of flurbiprofen, ibuprofen and ketoprofen.

The prodrug prepared having ester (Fig. **77**) and amide groups (Fig. **78**) like prodrugs of flurbiprofen, ibuprofen and ketoprofen [67]. These three non-steroidal anti-inflammatory drugs were esterified or amidated with five different alcohols or amines, respectively. They observed that the ester prodrugs were hydrolysed by human plasma with half lives ranging from 0.34 – 35.07 h. The data obtained reflect the utility of parallel combinatorial synthesis for the generation of simple prodrugs.

**Fig. (79):**
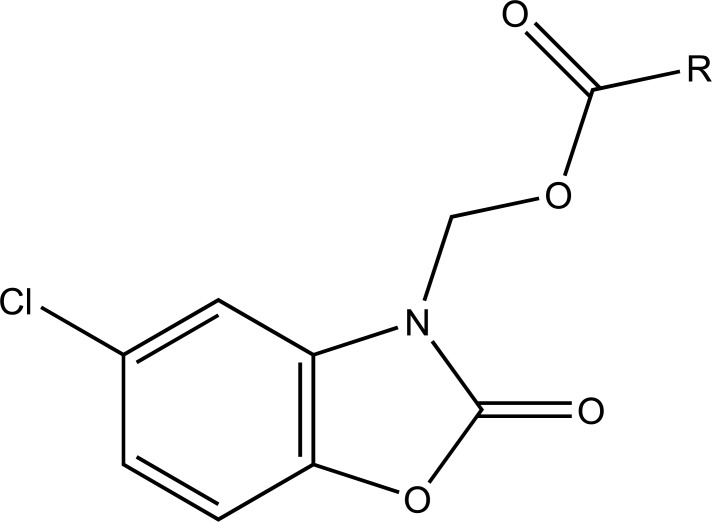
Chloroxazone ester prodrugs of some NSAIDs.

The prodrug was esterified with chloroxazone and some NSAIDs (ibuprofen, naproxen and mefenamic acid) (Fig. **79**) and evaluated for anti-inflammatory and muscle relaxant activities [68].

**Fig. (80):**
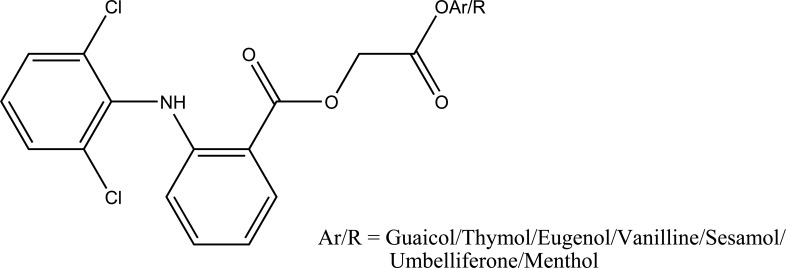
Diclofenac with different antioxidants.

The prodrugs of diclofenac were prepared using various antioxidants. It was found that diclofenac-antioxidant mutual prodrugs were reported as safer NSAIDs with lesser ulcerogenic toxicity [69]. The synthesized derivatives were screened for their antiinflammatory, analgesic and antiulcer activity. The synthesized mutual prodrugs showed retention of antiinflammatory activity with reduced ulcerogenic parameters. These results indicated that diclofenac-antioxidant mutual prodrugs (Fig. **80**) had the potential to develop better NSAIDs.

The prodrugs for aceclofenac were synthesized through the formation of amide linkage with methyl esters of amino acids like histidine (Fig. **81a**), alanine (Fig. **81b**), tyrosine (Fig. **81c**) and glycine (Fig. **81d**) [70]. The synthesized drug structures were elucidated by elemental analysis and different spectroscopic parameters. The *In vitro* studies were carried out to get the idea that the synthesized prodrugs would remain intact in simulated gastric fluid (SGF), simulated intestinal fluid (SIF) except simulated colonic fluid (SCF). In SCF, the enzyme amidase helps in the hydrolysis of the amide bond and releases free aceclofenac. The synthesized prodrug showed marked reduction in ulcer index and better anti-inflammatory activities.

**Fig. (82):**
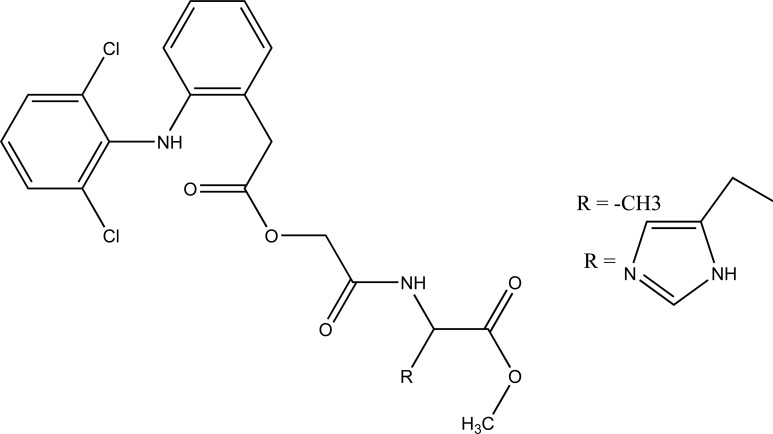
Aceclofenac with methyl esters of amino acids like histidine and alanine.

The prodrug formed by conjugation of aceclofenac with methyl esters of amino acids like histidine and alanine (Fig. **82**) [71]. These synthesized prodrugs were also screened for *in vitro* hydrolysis in SGF and SIF and in human plasma. The results obtained were alike of the previous experiment indicating that the prodrugs did not break in stomach, but release aceclofenac in intestine.

**Fig. (83):**
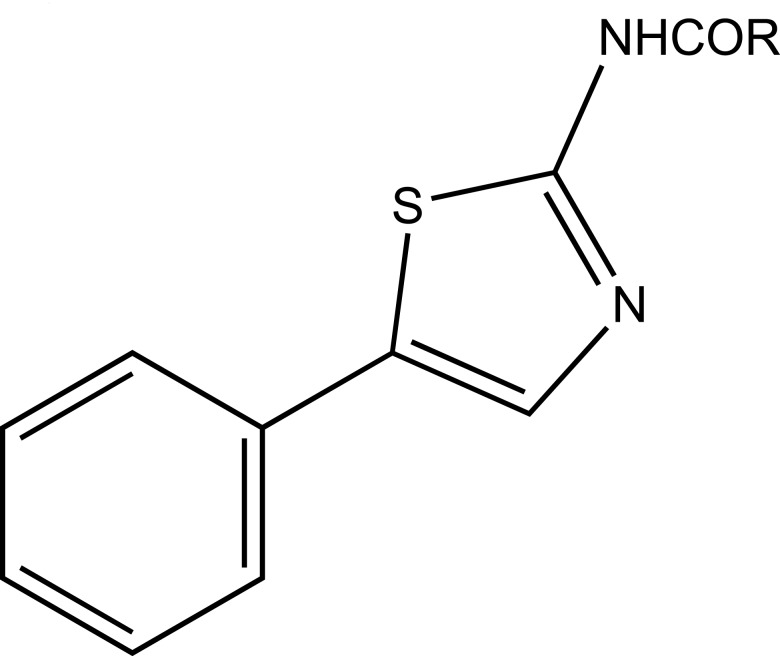
N-(5-phenylthiazol-2-yl) amides.

The prodrug synthesized by conjugating 2-amino-5-phenylthiazole with salicylic acid and the N-(5-phenylthiazol-2-yl) formed amides with ketoprofen, aceclofenac, flurbiprofen, mefenamic acid and indomethacin as illustrated (Fig. **83**). The conjugation of two structural motifs significantly increased the pharmacological activity. All compounds had shown reduction in ulcerogenic index.

**Fig. (84):**
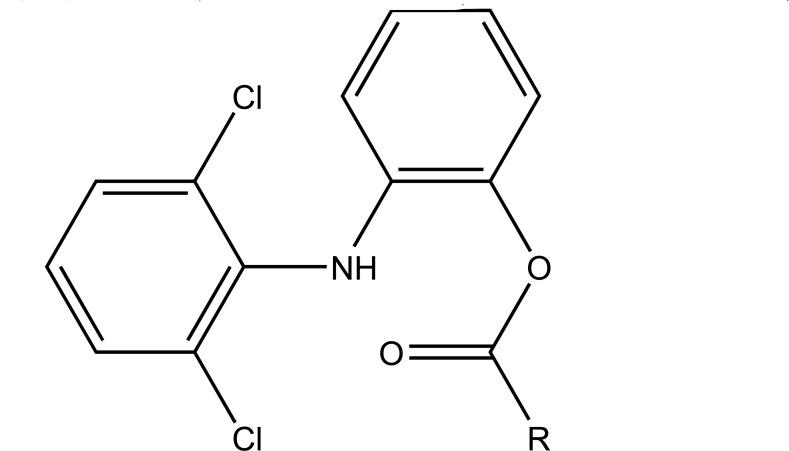
Amide prodrugs of diclofenac.

The prodrug of diclofenac was synthesized with amino acid derivatives (Fig. **84**) [72]. The amino acids selected were proline methyl ester, glutamic acid methyl ester, phenyl alanine methyl ester and sarcosine (2-methylglycine) ethyl ester. The parent compound was reacted with these derivatives. The result obtained clearly indicated that the synthesized prodrugs were found quantitatively less active than standard drug.

**Fig. (85):**
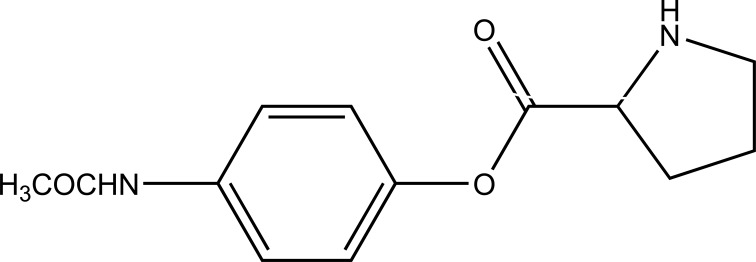
Proline ester prodrug of acetaminophen.

The prodrug of acetaminophen made with proline. (Pro-APAP) (Fig. **85**) and its hydrolytic studies were carried out in PBS buffer at various pH. The Pro-APAP was found to be stable at acidic pH than basic pH. The half-life of Pro-APAP at human plasma was found to be shorter.

**Fig. (86):**
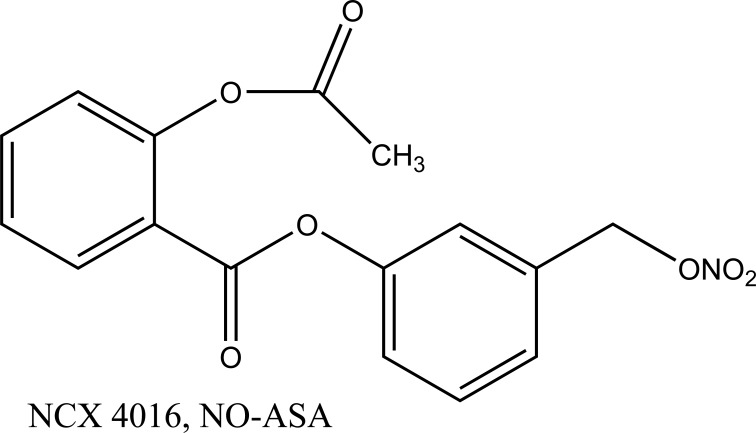
Nitrate [3-nitrooxyphenyl acetylsalicylate.

**Fig. (87):**
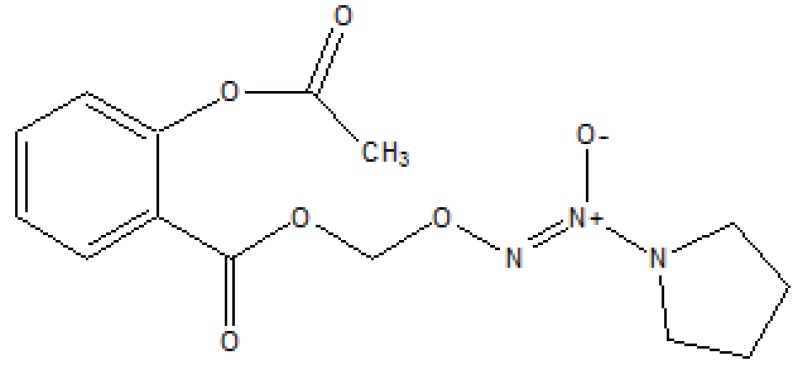
N-diazeniumdiolate.

An nitrate containing compound [3-nitrooxyphenyl acetylsalicylate (NO-ASA; NCX-4016)] (Fig. **86**) and an *N*-diazeniumdiolate [NONO-ASA, *O*_2_- (acetylsalicyloxymethyl)-1-(pyrrolidin-1-yl)diazen-1-ium-1,2-diolate (NONO-ASA; CVM-01)] (Fig. **87**), were quantified for ulcerogenic, anti-inflammatory, analgesic and antipyretic activity [73]. Both were found to be had same potency as analgesic and anti-inflammatory although superior than aspirin. However, they reduced PGE2 in stomach tissue so masked the gastric side effects.

**Fig. (88):**
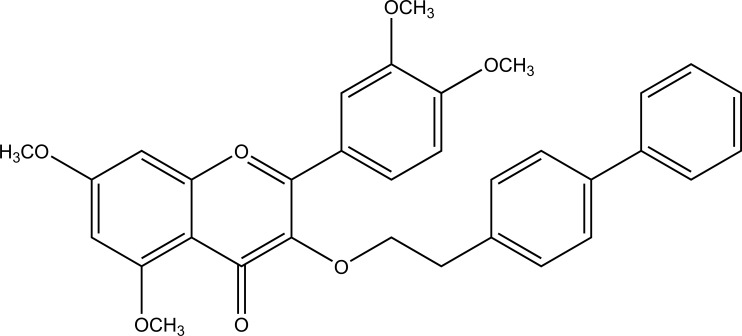
Mutual prodrug consisting of 4-biphenylacetic acid and quercetin tetramethyl ether.

A new mutual prodrug was prepared that had 4-biphenylacetic acid and quercetin tetramethyl ether (Fig. **88**) [74]. Its pharmacology and ulcerogenic studies were carried out. The results showed that the prodrugs formed had maximal lipophilicity and chemical stability. The synthesized compound also elicited comparable antiinflammatory activity with lesser ulcerogenicity.

**Fig. (89):**
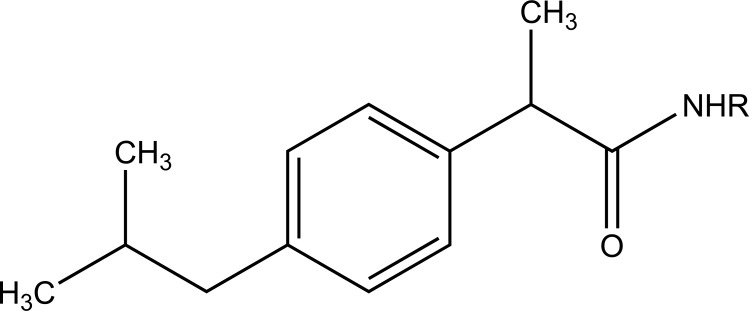
Amides of ibuprofen.

A series of ibuprofen amide prodrugs were prepared (Fig. **89**) with heteroaromatic amines and judged *in vivo* for their analgesic activity [75]. The result obtained showed synthesized prodrug showed good analgesic property and a lesser ulcerogenic activity.

**Fig. (90):**
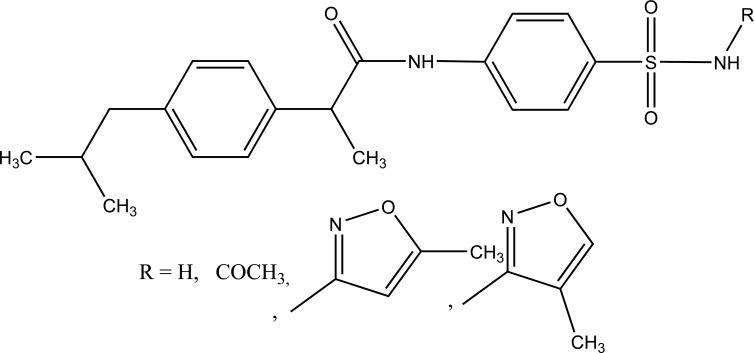
Ibuprofen with various sulfa drugs.

The prodrug of ibuprofen with various sulfa drugs was prepared. The prodrug was synthesized with the aim that it could be used for infection as well as for inflammation [76] (Fig. **90**).

**Fig. (91):**
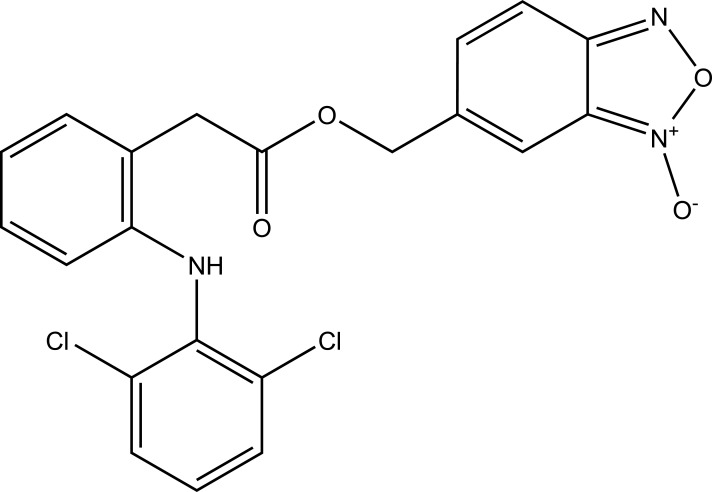
1-Oxy-benzo[1,2,5]oxadiazol-5-ylmethyl [2-(2,6-dichloro-phenylamino)-phenyl]-acetate, a new diclofenac derivative.

The prodrug was prepared with 1-Oxy-benzo [1, 2, 5]oxadiazol-5-ylmethyl [2-(2,6-dichloro-phenylamino)-phenyl]-acetate, a new diclofenac derivative (Fig. **91**) having a benzofuroxan heterocyclic moiety [77]. The pharmacological activity of this modified diclofenac was carried out that showed retention of the anti-inflammatory activity. The ulcerogenic properties of parent diclofenac were not seen in designed prodrug, although it showed the prevention of prostaglandin E_2_. The prodrug elicited good gastric tolerance.

**Fig. (92):**
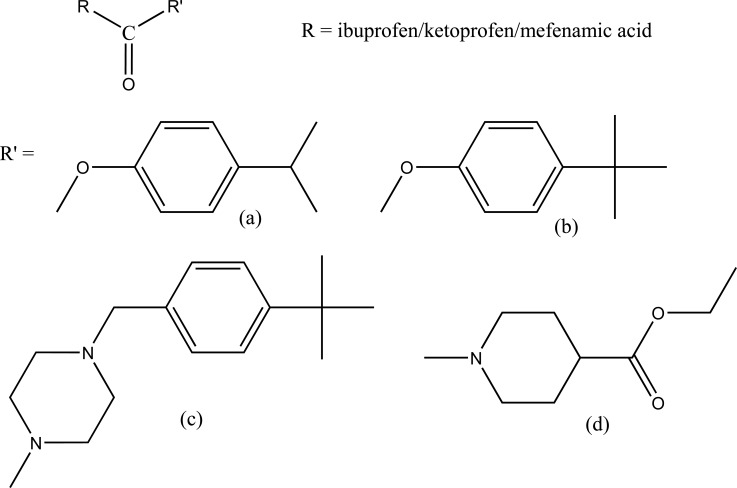
Ester and amide derivatives of some NSAIDs.

The ester and amide derivatives of ibuprofen, ketoprofen and mefenamic acid (Fig. **92a**; **b**; **c**; **d**) were synthesized and assessed for their analgesic and anti-inflammatory activity [78].

**Fig. (93):**
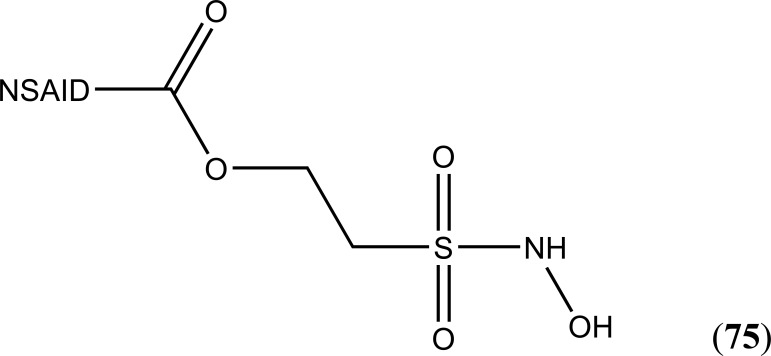
A group of hybrid ester prodrugs.

The prodrug was conjugated with the carboxylic acid group of indomethacin, (*S*)-naproxen and ibuprofen. It used a two-carbon ethyl spacer and a sulfohydroxamic acid component (CH_2_CH_2_SO_2_NHOH) to equip a group of hybrid ester prodrugs (Fig. **93**) that released nitric oxide (NO) and nitroxyl (HNO) moiety [79]. All compounds showed remarkable NO, but same HNO. The prodrugs of (*S*)-naproxen and ibuprofen were influential and relatively more active than parent drugs. The prodrug of indomethacin was found to be less ulcerogenic than indomethacin itself. It was presumed that the synthesized prodrug acts like selective COX-2 inhibitor with retain anti-inflammatory activity.

**Fig. (94):**
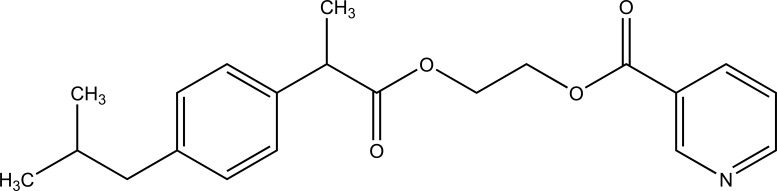
Codrug of nicotinic acid and ibuprofen.

The prodrug was synthesized with nicotinic acid and ibuprofen (Fig. **94**) with the aim to mask the possible side effects of nicotinic acid. This is done by making the ester of these. This blocks the synthesis of prostaglandin and released ibuprofen [80]. Prodrug and ibuprofen 2-hydroxyethyl ester elicited improved *in vitro* enzymatic hydrolysis than chemical hydrolysis.

**Fig. (95):**
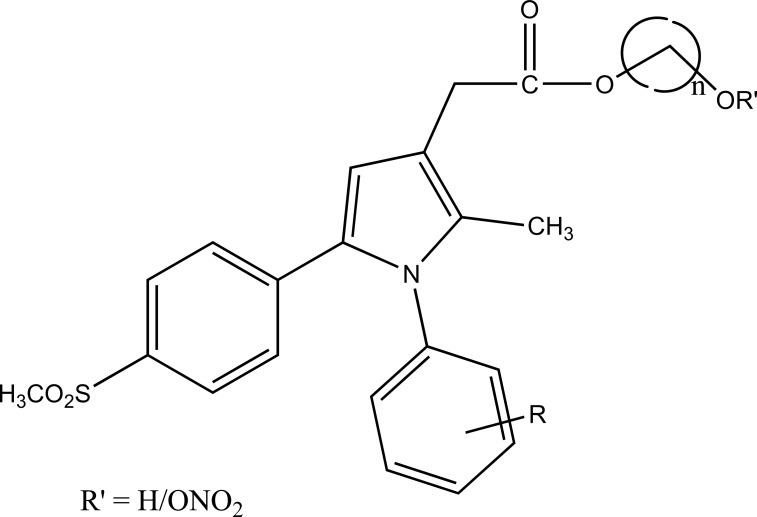
Pyrrole-derived nitrooxy esters.

A novel series of pyrrole-derived nitrooxy esters were synthesized. They were designed in such a way that they were exhibiting cyclooxygenase (COX) inhibition and maintain the release of nitric oxide (NO). This would lead to develop safer drug for the gastric and cardio related troubles [81]. (Fig. **95**). The synthesized prodrugs were selective regulator of cyclooxygenase-2 (COX-2) with NO releasing properties and reduced related adverse side effects.

**Fig. (96):**
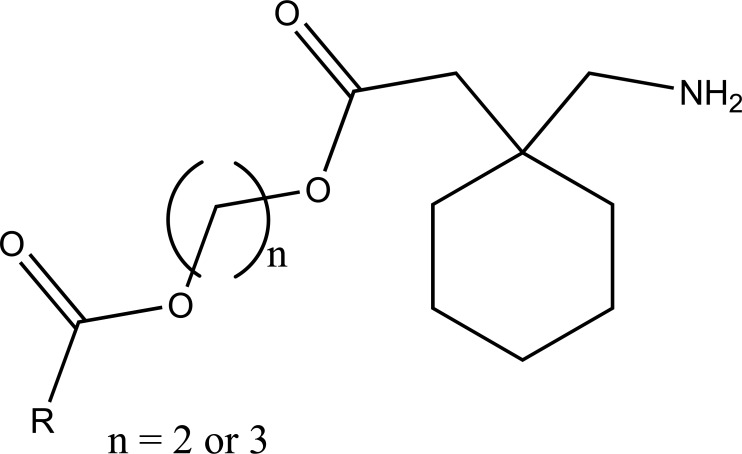
NSIADs with gabapentin via ester bonds.

The prodrug of some NSIADs with gabapentin was prepared via ester bonds (Fig. **96**). It used glycol spacers to reduce the gastric adverse effects and getting synergistic analgesic effects [82].

The aminoethyl (Fig. **97a**) and aminobutyl esters (Fig. **97b**) of ketorolac were prepared. They had 1-methylpiperazine, N-acetylpiperazine or morpholine substituents. Its hydrolysis kinetics study was done [83]. The hydrolysis data revealed that the aminobutyl esters are the most stable.

The prodrug was synthesized with two derivatives (Fig. **98a**; **b**) of compounds consisting of mefenamic acid, glycine and organic nitrates (2-nitrooxy ethanol or 1,3-dinitrooxy-2-propanol) [84]. The Nitric oxide (NO) caused mucosal protection mechanisms as prostaglandins which was responsible for ulcer healing. This gave the idea that these compounds would reduce NSAIDs associated GI side effects.

The esters of ibuprofenic acid and mefenamic acid, named as 4-((4-substituted benzylidene)amino)phenyl 2-(4-isobutylphenyl) propanoate (Fig. **99a**) and 4-((4-substituted benzylidene) amino) phenyl 2-((2,4-dimethylphenyl)amino)benzoate (Fig. **99b**) analogs were synthesized by fusion with the respective acids via use dicyclohexyl carbodiimmide [85]. The prime aim was to eradicate the problem of GI toxicity due to NSAIDs use. Pharmacological and ulcerogenic properties of the synthesized prodrugs were judged *in vivo* and correlated with that of parent drug.

**Fig. (100):**
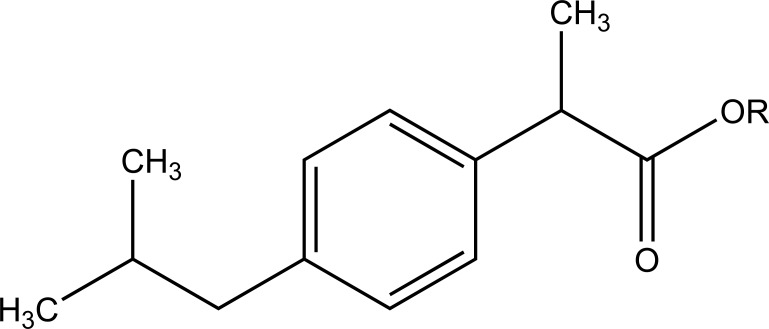
Ibuprofen with naturally occurring phenolic and alcoholic compounds.

The mutual prodrug of ibuprofen was synthesized with naturally existing components bearing phenol and alcohol groups like menthol, thymol and eugenol and evaluated for pharmacological activities (Fig. **100**) [86]. Here, naturally occurring phenolic and alcoholic compounds were chosen with the target of getting synergistic effect.

**Fig. (101):**
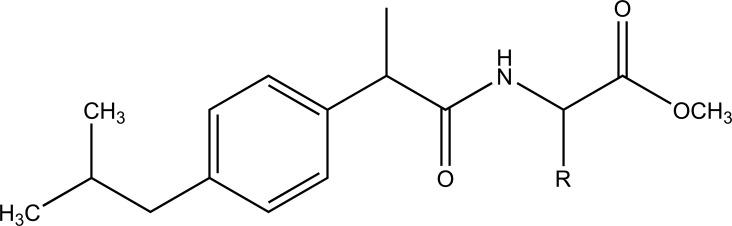
Prodrugs of dexibuprofen.

The prodrugs of dexibuprofen were synthesized (Fig. **101**) having ester and amide linkage [87]. The acid chloride of Dexibuprofen synthesized via variety of methyl ester hydrochlorides of amino acid and five alcohols to have the amide and ester prodrugs. The kinetics study showed that the synthesized prodrugs were less GI toxicity than dexibuprofen.

**Fig. (102):**
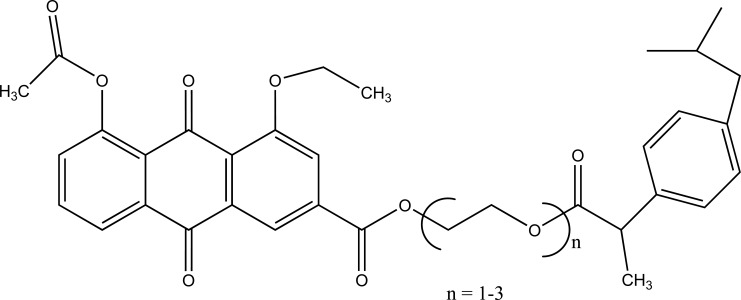
Rhein-NSAIDs prodrugs.

The rhein-NSAIDs prodrugs were synthesized (Fig. **102**) through glycol esters that showed significant anti-inflammatory activity and possessed less degree of ulcerogenic potential [88].

**Fig. (103):**
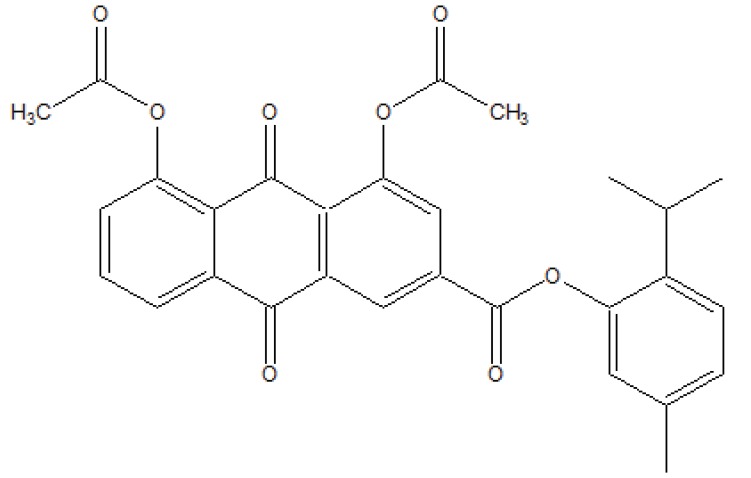
Co-drug of diacerein with antioxidant thymol.

The prodrug (Fig. **103**) designed and synthesized with diacerein and antioxidant thymol [89]. The hydrolysis kinetics studies were performed in phosphate buffer (pH 7.4) and small intestine. The synthesized prodrug had improved lipophilicity and hence bioavailability. The data indicated it was used as a drug used in the management of osteoartiritis.

**Fig. (104):**
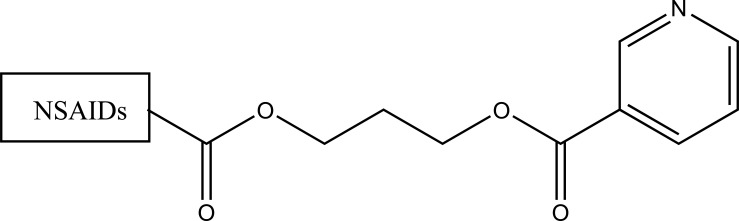
Nicotinic acid conjugates with NSAIDs.

The prodrug was synthesized with a series of nicotinic acid conjugates (Fig. **104**) with NSAIDs using (*O*-(Benzotriazol-1-yl)-*N*, *N*, *N*′, *N*′-tetramethyluronium tetrafluoroborate) in good yield [90]. All the synthesized drugs were evaluated for their *in vitro* anti-inflammatory activity.

**Fig. (105):**
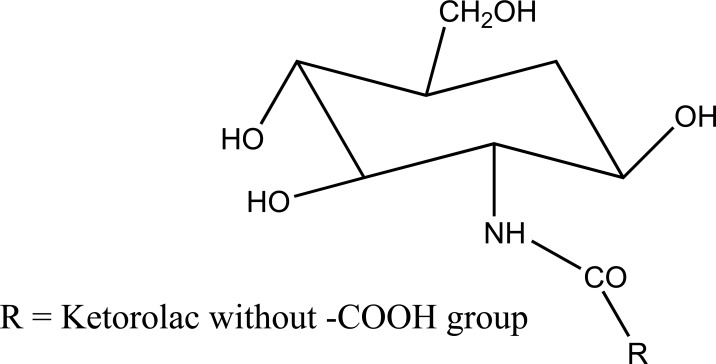
Mutual amide prodrug of ketorolac with glucosamine.

The amide prodrug of ketorolac was synthesized with (Fig. **105**) glucosamine [91]. *In vitro* hydrolytic studies of prodrug showed good rate of hydrolysis in blood plasma and simulated intestinal fluid whereas it was stable in gastric simulated fluid. *In vivo* pharmacological studies were done on animals, elicited good analgesic, antiinflammatory and antiarthritic activity. The data showed better action of prodrug as compared to ketorolac and showed less gastrointestinal side effects. The prodrug was evaluated by ulcerogenic and histopathologic analysis. Histopathological study showed less ulceration in the gastric region.

**Fig. (106):**
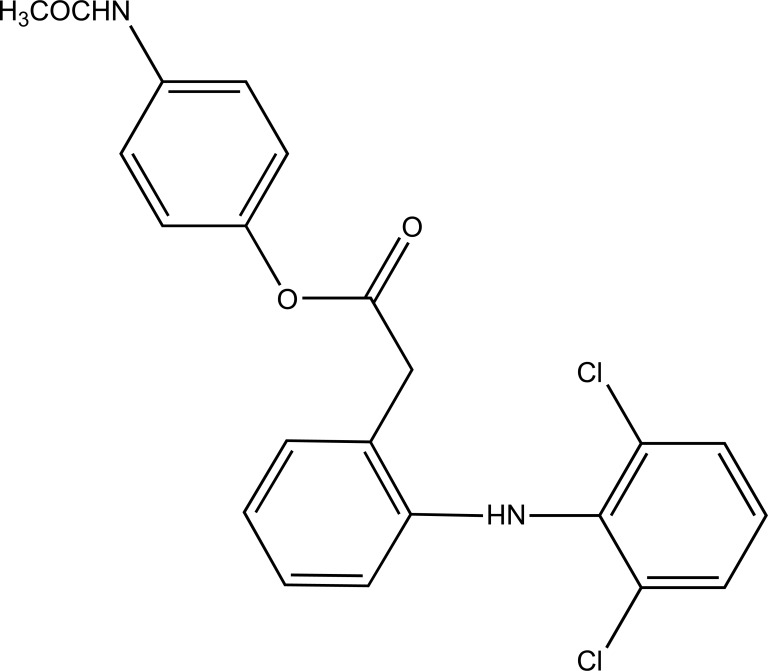
Mutual prodrug of diclofenac and paracetamol.

The mutual prodrug (Fig. **106**) of diclofenac and paracetamol was prepared and carried out there *in vitro* hydrolysis studies [92]. The purity of the prodrug was confirmed by TLC and characterized on the basis of IR spectroscopy and ^1^H NMR spectroscopy. The physiochemical parameters were determined and the results showed that they were more lipophilic than the parent drug. The compound was also evaluated for anti-inflammatory and ulcerogenicity.

**Fig. (107):**
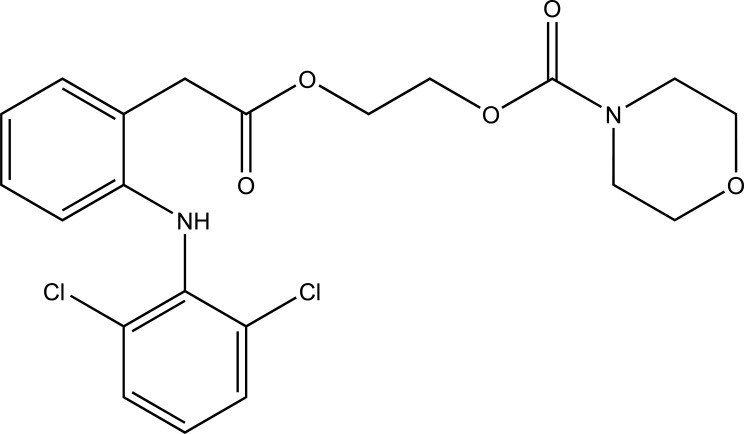
Morpholine ester of diclofenac.

**Fig. (108):**
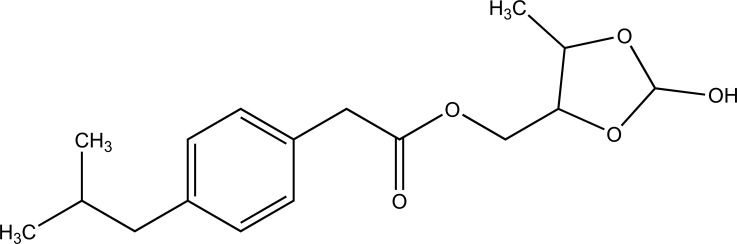
Medoxinil prodrug of ibuprofen.

The prodrug synthesized N-ethoxycarbonylmorpholine ester of diclofenac (Fig. **107**) [93]. The stability of the prodrug was evaluated in buffer solution and in plasma. The synthesized prodrug showed retention of anti-inflammatory activity and reduction in ulcerogenecity at gastrointestinal tract than the drug diclofenace sodium. The prodrug is synthesized with medoxomil prodrug of ibuprofen (Fig. **108**) and carried out their in vitro hydrolysis studies [94]. The physical and chemical parameters were calculated and the results showed that they had improved oral bioavailability, reduced irritation, and prolonged action time than the parent drug. Prodrug showed high stability in gastrointestinal tract juice and rapid hydrolysis in plasma.

**Fig. (109):**
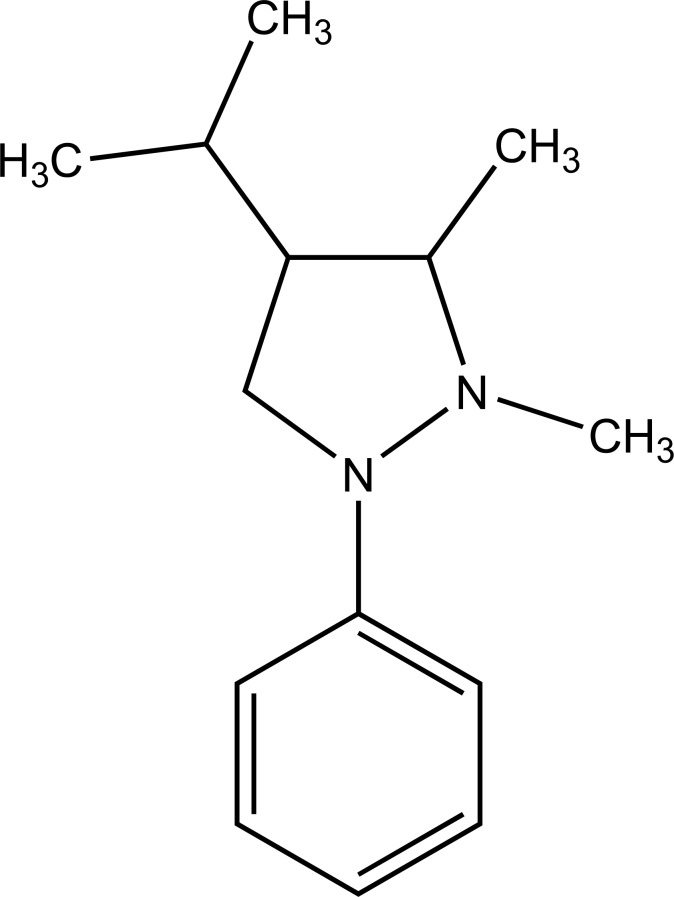
Propylphenazone with acidic NSAIDs.

**Fig. (110):**
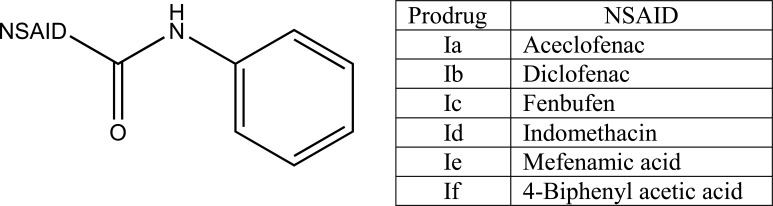
Amide based prodrugs.

The prodrug is coupling of propyphenazone (Fig. **109**) with acidic NSAIDs such as ketoprofen, ibuprofen, and diclofenac produced mutual prodrug with synergistic effects [95]. Propyphenazone was a nonacidic pyrazole NSAID that has analgesic effect with minimal anti-inflammatory activity. Mutual prodrug evaluated both *In Vitro* and *In Vivo* and it also showed minimal GI irritation. The prodrug is synthesized with a total of six amide based prodrugs (Ia-f) (Fig. **110**) of aceclofenac, diclofenac, fenbufen, indomethacin, mefenamic acid and 4-biphenyl acetic acid through one-pot method (single step method) [96]. Hydrolysis of prodrug was studied by reverse phase HPLC method in acidic buffer (hydrochloric acid) (pH 1.2) and phosphate buffer (pH 7.4). The prodrug is evaluated for their ulcerogenic activity and anti-inflammatory activity and compared to their corresponding drugs.

## CONCLUSION

This review is based on literature of prodrugs that protects gastric toxicity. This opens the field of research towards design of drugs through prodrug approach. It develops the idea that a lot many researchers are involved in developing safer NSAIDs. This literature would help the scientist to get the preliminary idea of the research done in the field of prodrugs as NSAIDs. This helps to generate the view that development of NSAIDs as prodrug is required due to the side effects they have. This paper would lead to generate the idea how the safer NSAIDs can be developed which may be fruitful for the society.

## Figures and Tables

**Fig. (26a):**
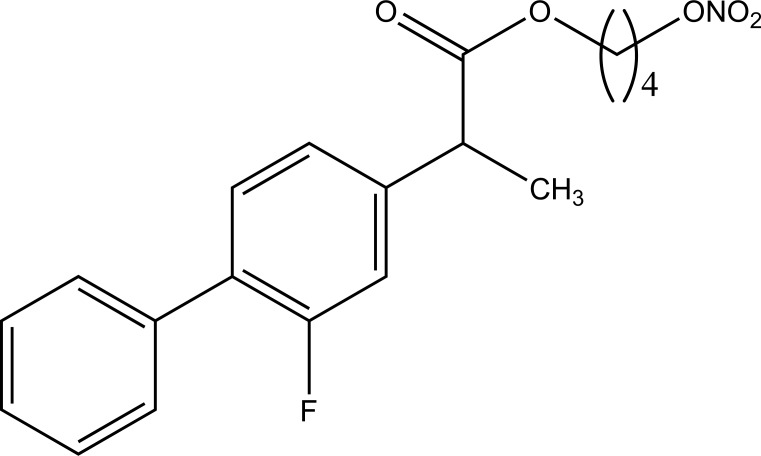
Nitroxybutylesters of flubiprofen.

**Fig. (26b):**
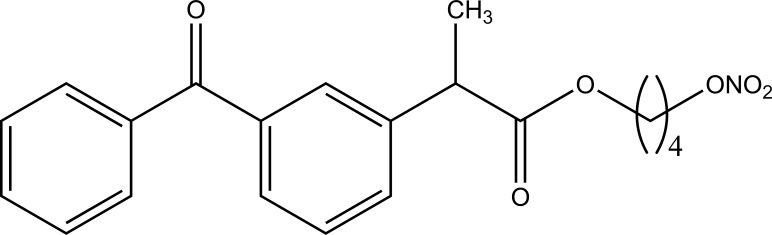
Nitroxybutylesters of ketoprofen.

**Fig. (32a):**
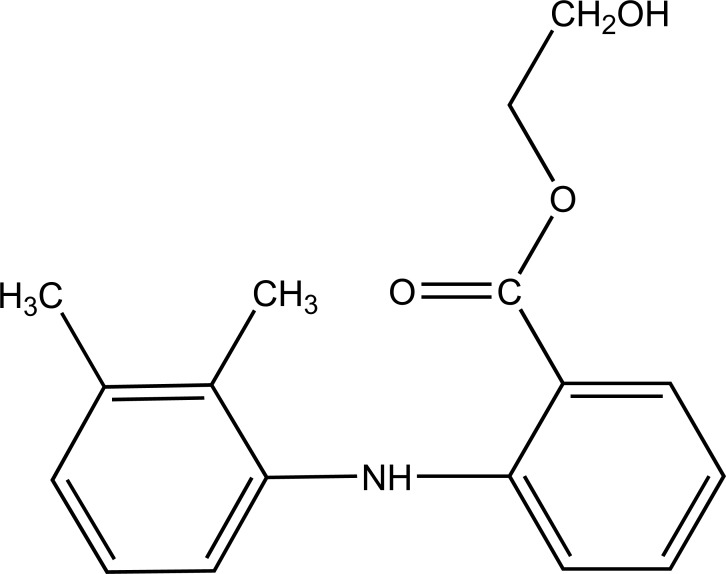
Hydroxyethyl esters of mefenamic acid.

**Fig. (32b):**
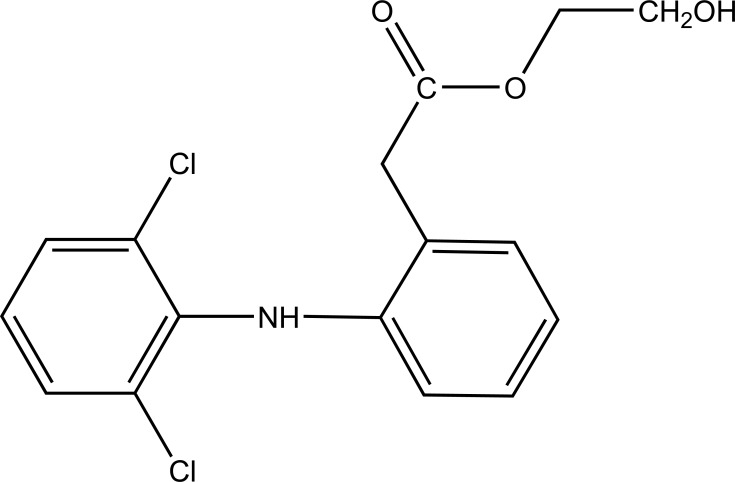
Hydroxyethyl esters of diclofenac.

**Fig. (39a):**
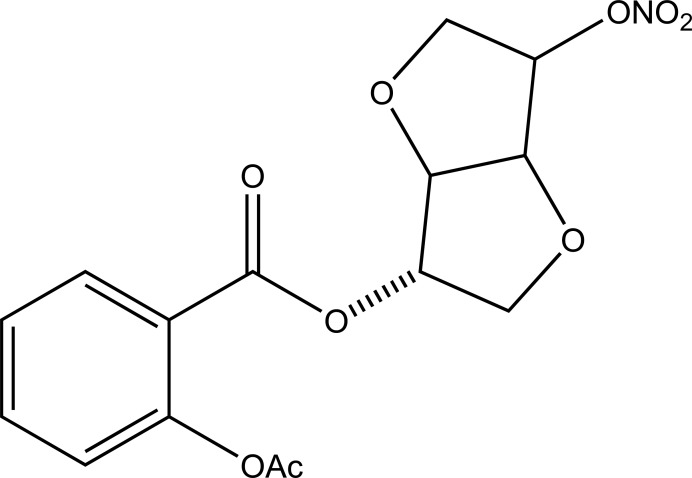
First isomeric aspirin derivatives of isosorbide-5-mononitrate.

**Fig. (39b):**
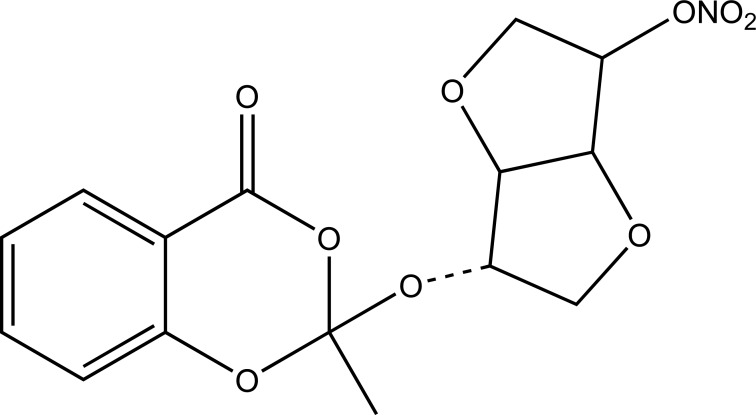
Second isomeric aspirin derivatives of isosorbide-5-mononitrate.

**Fig. (43a):**
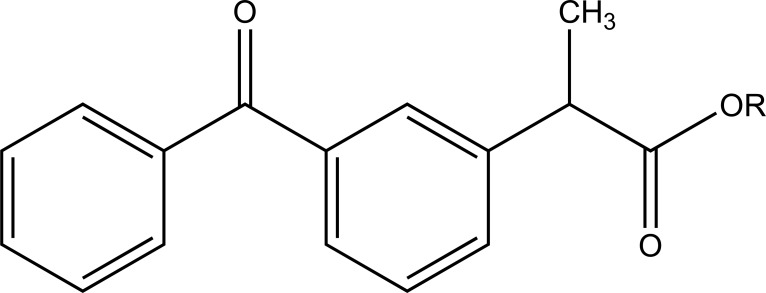
Ketoprofen prodrug.

**Fig. (43b):**
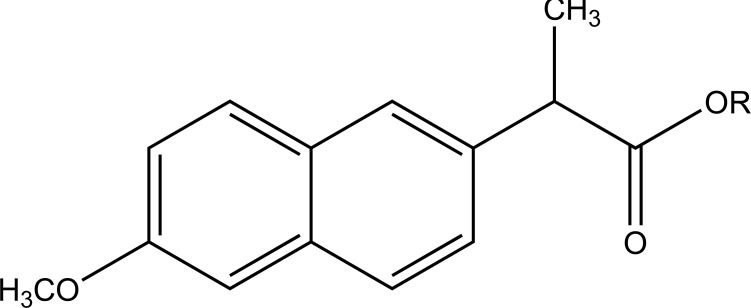
Naproxen prodrug.

**Fig. (43c):**
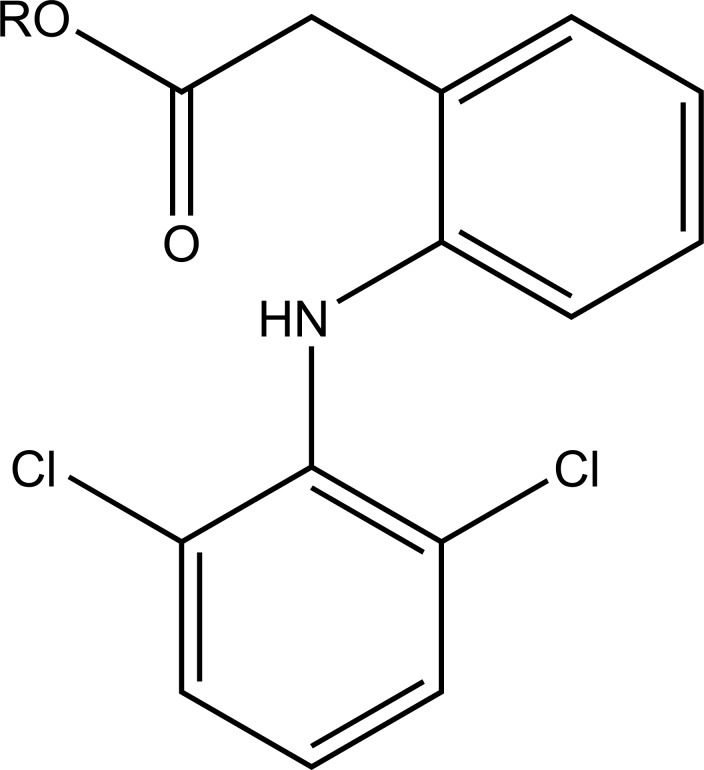
Diclofenac prodrug.

**Fig. (44a):**
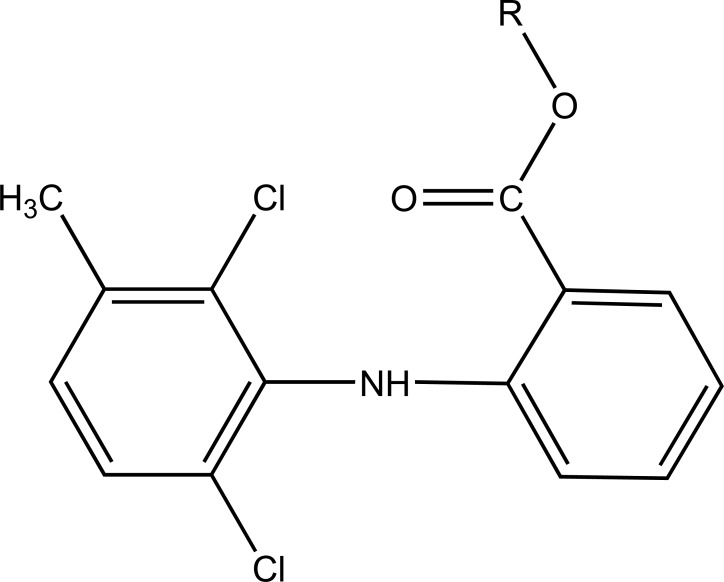
Amides of meclofenamic acid.

**Fig. (44b):**
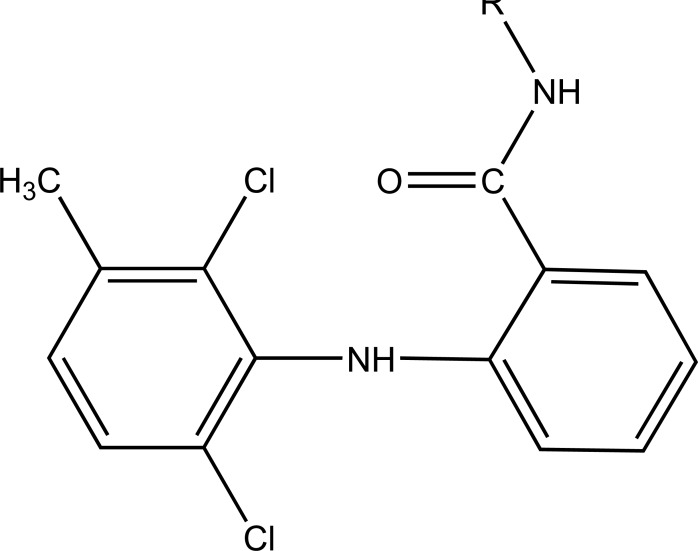
Amides of meclofenamic acid.

**Fig. (74a):**
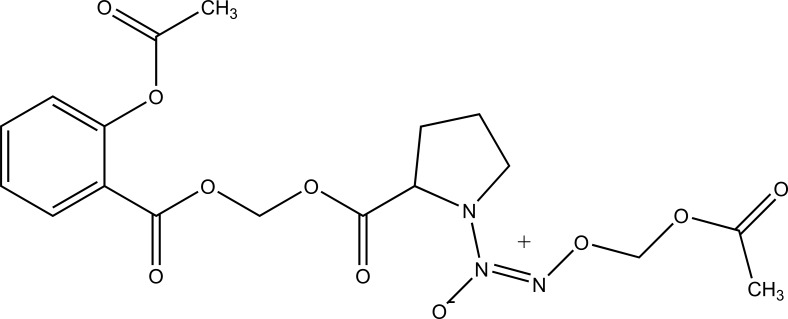
Aspirin prodrug.

**Fig. (74b):**
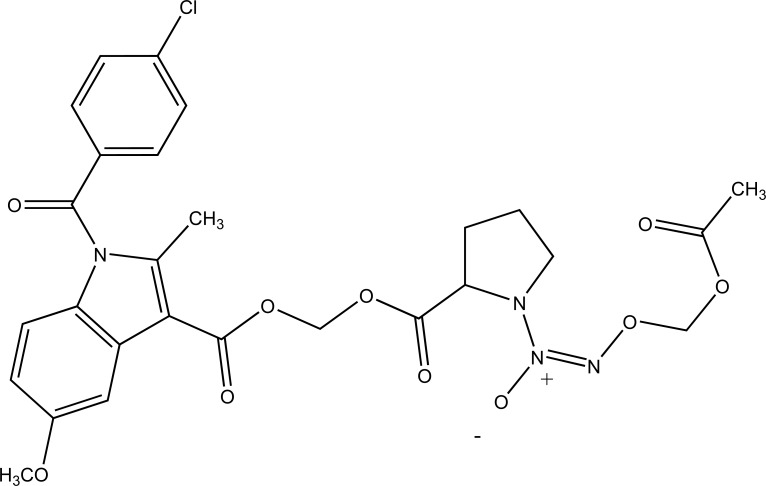
Indomethacin prodrug.

**Fig. (81a):**
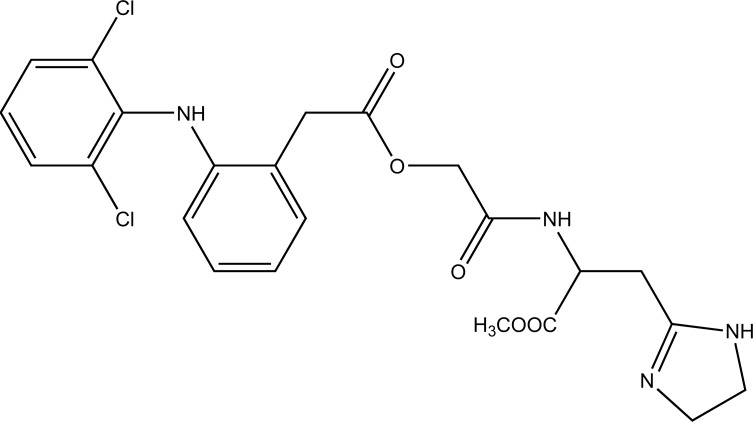
Methyl esters of amino acids like histidine.

**Fig. (81b):**
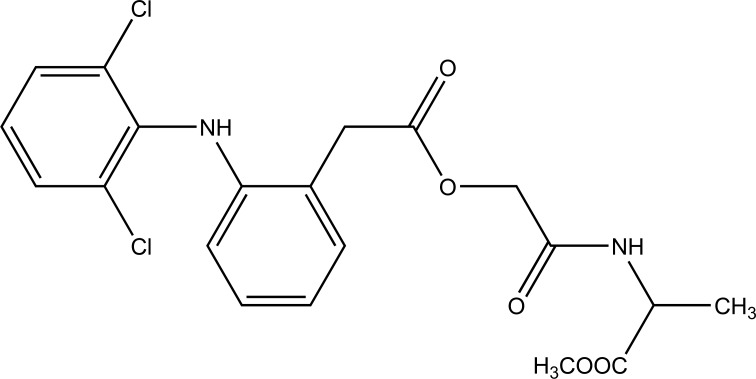
Methyl esters of amino acids like alanine.

**Fig. (81c):**
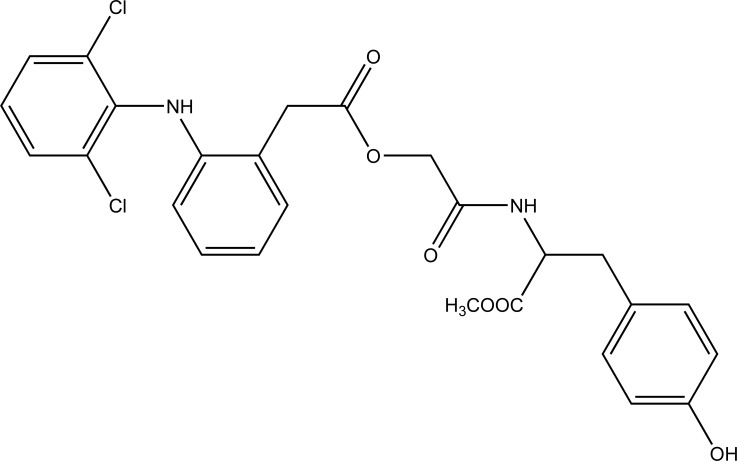
Methyl esters of amino acids like tyrosine.

**Fig. (81d):**
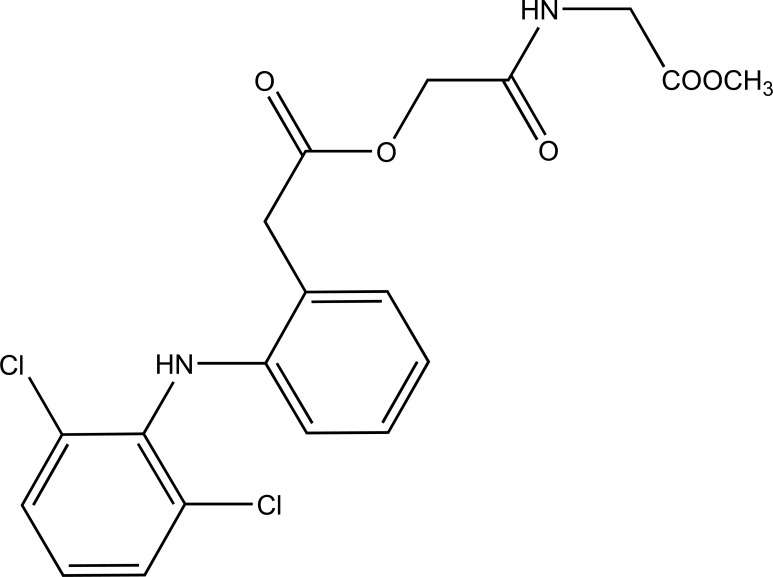
Methyl esters of amino acids like glycine.

**Fig. (97a):**
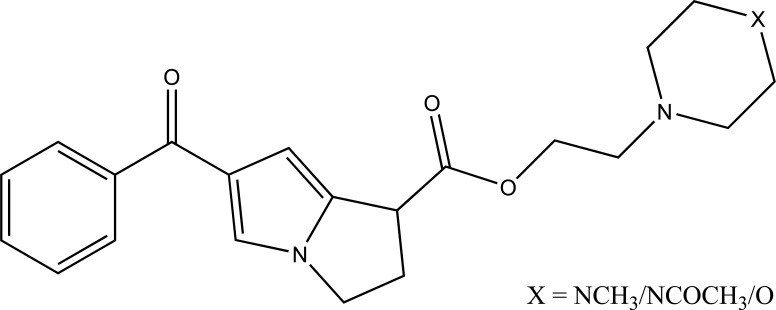
Aminoethylesters of ketorolac.

**Fig. (97b):**
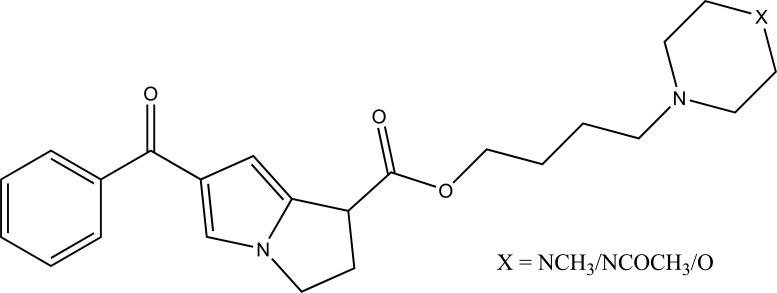
Aminobutyl esters of ketorolac.

**Fig. (98a):**
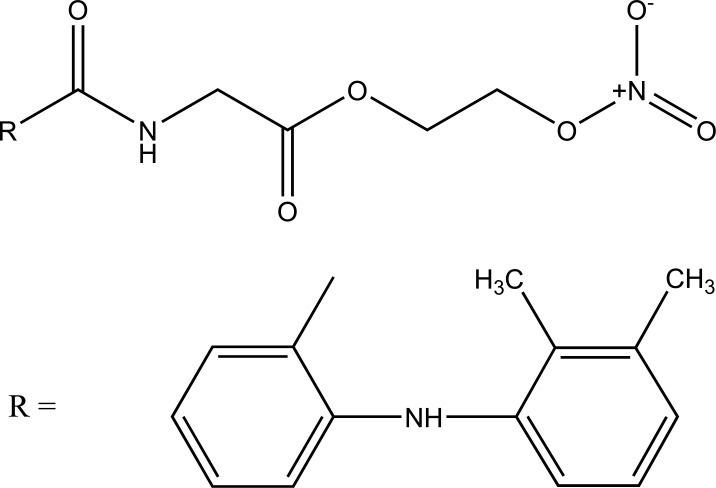
Mefenamic acid, glycine and organic nitrates.

**Fig. (98b):**
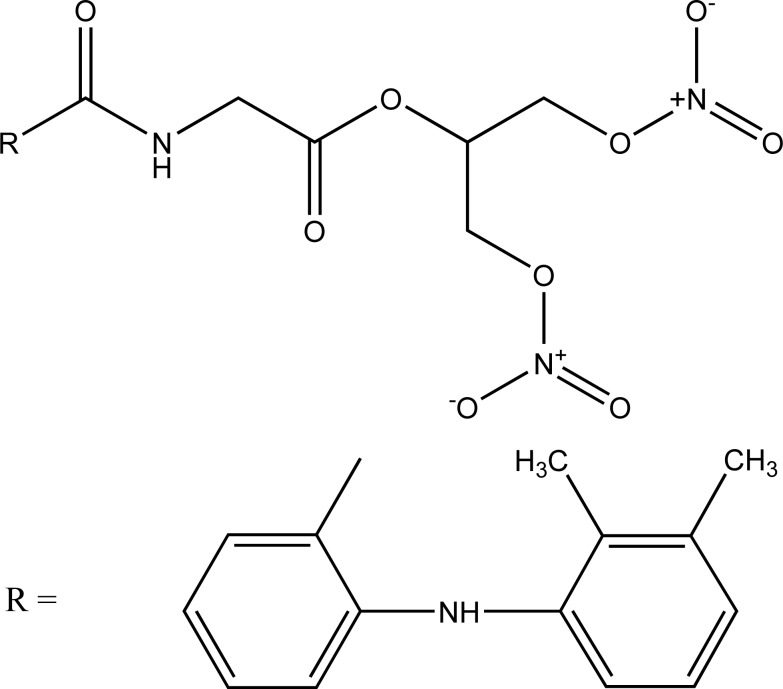
Mefenamic acid, glycine and organic nitrates.

**Fig. (99a):**
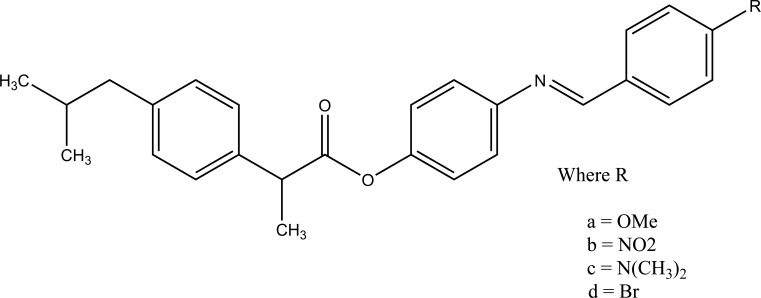
4-((4-substituted benzylidene)amino)phenyl 2-(4-isobutylphenyl) propanoate.

**Fig. (99b):**
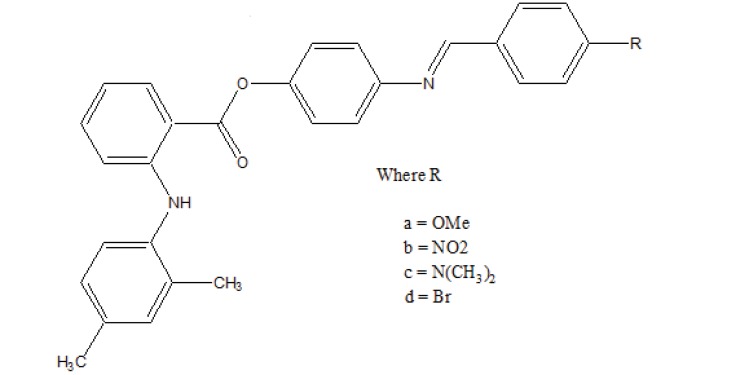
4-((4-substituted benzylidene)amino)phenyl 2-((2,4-dimethylphenyl)amino)benzoate.

**Table 1:** Examples of prodrug and the purpose of modification.

**Parent Drug**	**Prodrug**	**Reason of Modification**
Amoxicillin	Sarmoxicillin	Increase distribution
Ampicillin	Bacampicillin, Pivampicillin, Talampicillin	Increase distribution
Ampicillin	Hetacillin Enhance Bioavailability	Increase stability
Carbencillin	Carfecillin, Carindacillin	Enhance Bioavailability
Chloramphenicol	Chloramphenicol palmitate ester	Improve Taste
Chloramphenicol	Chloramphenicol succinate ester	Water solubility
Clindamycin	Clindamycin palmitate ester	Improve taste
Clindamycin	Clindamycin 2’ phosphate ester	Decrease pain on injection
Cefamandole	Cefamandole nafate ester	Stability
Cycloserine	Pentizidone	Stability
Diethylstilbesterol	Fostestrol	Decrease gastric distress
Dopamine	L-dopa	Delivery to brain
Epinephrine	Dipirefrin	Corneal penetration
Erythromycin	Erythromycin ethylsuccinate	Gastric stability
Estradiol	Estradiol cypionate	Extend duration
Elilefrine	Elilefrine stearate ester	Bioavailability
Fluphenazine	Fluphenazine decanoate	Long acting depot injections
Formaldehyde	Methenamine	Urinary tract delivery
Metronidazole	Amino acid esters,	Water solubility
	Benzoyl derivative	Mask taste
Naloxone	Mono and disulphate ester	Extend duration
Nitrogen Mustard	Amide derivative	Delivery to neoplastic tissue
Salicylic acid	Salsalate	Gastrointestinal tolerance and bioavailbility
Sulfisoxazole	Acetyl esters	Improve taste
Testosterone	Testosterone propionate	Extend duration
Triamcinolone	Acetonide	Increase topical activity
